# Psychiatric health of Icelandic adults 40 years or older: A nationwide study of diagnoses, medications, and symptoms

**DOI:** 10.1371/journal.pone.0342075

**Published:** 2026-04-15

**Authors:** Inga Wessman, Lauren P. Wadsworth, Lárus Steinþór Guðmundsson, Þorvarður Jón Löve, Sæmundur Rögnvaldsson, Guðmundur Bjarni Arnkelsson, Andri Ólafsson, Sigrún Þorsteinsdóttir, Björn Leví Gunnarsson, Guðrún Ásta Sigurðardóttir, Nicholas J. Sibrava, Ásdís Rósa Þórðardóttir, Gauti Kjartan Gíslason, Tinna Laufey Ásgeirsdóttir, Sigurður Yngvi Kristinsson, Andri S. Bjornsson

**Affiliations:** 1 Department of Psychology, University of Iceland, Reykjavík, Iceland; 2 Genesee Valley Psychology, Rochester, New York, United States of America; 3 Department of Pharmaceutical Sciences, University of Iceland, Reykjavík, Iceland; 4 Department of Medicine, University of Iceland, Reykjavík, Iceland; 5 Landspítali – The National University Hospital of Iceland, Reykjavík, Iceland; 6 Rigshospitalet, Copenhagen, Denmark; 7 Baruch College – City University of New York, New York, New York, United States of America; 8 Department of Economics, University of Iceland, Reykjavík, Iceland; Istanbul Bakirkoy Prof Dr Mazhar Osman Ruh Sagligi ve Sinir Hastaliklari Egitim ve Arastirma Hastanesi, TÜRKIYE

## Abstract

Nationwide, population-based psychiatric health data, preferably incorporating information from various assessment methods such as health registries and self-report measures, are needed to manage healthcare systems and shape public health policies. The aim of the study is to assess the psychiatric health of individuals 40 years and older by examining data on psychiatric diagnoses, medication, anxiety and depressive symptoms, and life satisfaction. This is a cross-sectional study using a nationwide cohort. All Icelanders 40 years and older were invited to participate in this cross-sectional study, and 80,759 (54.3%) registered. Data from health registries and validated self-report measures were used. A total of 16,764 individuals (20.8%) received one or more psychiatric diagnoses in the year prior to starting the study, with nonorganic sleep disorders, other anxiety disorders, and a depressive episode being the most common. Additionally, 27,642 (34.2%) filled at least one psychotropic prescription (19.9% without a formal diagnosis filled prescriptions), with 24.3% filling one or more prescriptions for benzodiazepine related medications or benzodiazepine derivatives. Rates of diagnoses and filled prescriptions increased across age groups. Most participants reported no or mild anxiety (94.3%), and minimal or mild depressive symptoms (88.8%). Over half (59.3%) reported satisfaction or extreme satisfaction with life. The results reveal the widespread prevalence of psychiatric disorders across the population in a real-world, nationwide context, emphasizing the need for psychiatric care. However, the non-specific and broad nature of diagnoses limits the ability to provide appropriate care. Additionally, the findings highlight a reliance on psychotropic medications to manage psychiatric symptoms and disorders, even without a formal diagnosis. The rate of filled benzodiazepine prescriptions is alarming, as these medications are not recommended as a first-line treatment for psychiatric disorders. The findings provide population-based psychiatric health data that can inform healthcare management and guide public health policy.

## Introduction

Psychiatric disorders are a major contributor to the global burden of disease, having increased in rank from the ninth to the sixth leading cause of health loss worldwide between 1990 and 2021 [1,2]. Accurate, nationwide, population-based psychiatric health statistics are essential for effective healthcare system management and public health policy, ideally incorporating data from multiple assessment methods, including health registries and self-report measures. Current global statistics on psychiatric health are based on data gathered using non-comprehensive assessment methods, with reliance on population-based surveys [3]. Population-based surveys frequently underrepresent individuals with prior psychiatric diagnoses [4], as well as individuals in institutional settings and males or younger adults [3], which may lead to an underestimation of psychiatric health care needs at the population level.

According to the Institute for Health Metrics and Evaluation (IHME; estimates are generated through statistical modeling that integrates data from population-based surveys, epidemiological studies, and healthcare registries), 13% of the world population suffered from a psychiatric disorder (excluding dementia, substance use disorders, and other less common disorders) in 2019 [1], increasing to 14% in 2021 [2]. In addition, according to the World Health Organization, 7% suffered from alcohol use disorder in 2019 [5], 0.5% from drug use disorder in 2021 [6], and 8% of individuals 65 years or older from dementia in 2019 [7]. However, reported prevalence estimates for psychiatric disorders vary widely across different reports, studies, and meta-analyses, ranging from 13−41%. The variability is at least partly due to differences in research designs, assessment methods (for example, surveys, self-report measures assessing psychiatric symptoms, diagnostic interviews, or registry data), inclusion and exclusion criteria (for example, inclusion or exclusion of childhood onset disorders, dementia, or substance use disorders), and age group coverage (for example, all age groups, 18 years or younger, 18 years or older, or a specific age group like older adults) [8,9].

Rates of psychotropic medication use also vary. According to one study published 20 years ago, 12% of Europeans reported taking at least one psychotropic medication on one or more occasions (i.e., antidepressants, anxiolytics, antipsychotics, and mood stabilizers) in the previous year, with a range of 6−19% depending on the country [10]. Recent data from Europe indicate that psychotropic medication use remains within the range reported in the earlier study, with 13% of individuals in England [11] and 14% in Spain reporting current use [12]. In the United States, 17% of adults reported taking at least one psychotropic medication in 2020, with medication use being more common among older adults (i.e., 18% of individuals aged 45−64 years old and 17% of individuals aged 65 and older compared to 15% of individuals aged 18−44 years old) [13]. Updated psychiatric health statistics are needed for psychiatric diagnoses and psychotropic medication use across time.

Overall, residents of European Union countries report satisfactory or higher levels of life satisfaction [14]. In most member states, life satisfaction is greater among younger adults (16–29 years old) than older adults, although in Denmark, Sweden, the Netherlands, Luxembourg, and Finland, older adults (65 years and older) report greater satisfaction than younger adults [14]. In northern European countries, including Iceland, the traditional U-shaped association between life satisfaction and age (typically characterized by higher satisfaction in young adulthood, a decline through midlife, and a rise again in older age) has shifted toward a pattern of increasing satisfaction with older age [15]. Longitudinal evidence further shows that life satisfaction remains high and relatively stable over time, with only a weak to moderate association with mental health, suggesting that many individuals maintain satisfaction with life despite changes in mental health [16]. Despite this paradox, limited data on subjective measures such as life satisfaction alongside objective measures of psychiatric health (e.g., psychiatric disorder diagnoses and psychotropic medications) are available.

Previous reports and studies have mostly involved either empirical studies with small subsets of the relevant populations and non-comprehensive assessment methods (e.g., self-reported psychiatric diagnosis or medication use) or systematic reviews based on a mixture of empirical studies. To our knowledge, the current study is among the most comprehensive nationwide population-based studies measuring psychiatric health in middle-aged and older adults within a single country. The study provides unique, comprehensive statistics on psychiatric health derived from multiple data-collection methods (e.g., medical registries and self-report measures, including satisfaction with life) and draws on a large proportion of data collected in a real-world setting. Comparing these measures within a single population provides a more accurate picture of the psychiatric health among individuals 40 years and older.

## Method

### Participants

This is a cross-sectional study using a nationwide cohort. All residents of Iceland 40 years and older (148,096) were invited to participate. The study is part of a randomized controlled trial screening for a precursor of multiple myeloma (MM), monoclonal gammopathy of undetermined significance (MGUS), as previously described [17]. The population was restricted to individuals aged 40 years and older, as MGUS is rare in younger adults, and the primary objective of the overall study is to investigate MGUS and its progression to MM. In total, 80,759 (54.3%) individuals registered for the study between September 15^th^, 2016, and February 20^th^, 2018, however, 26 participants withdrew consent prior to the current study.

### Instruments

#### The Generalized Anxiety Disorder Scale (GAD-7).

GAD-7 [18] is a seven-item self-report scale used to measure the severity of anxiety symptoms in the past two weeks with good to excellent internal consistency, test-retest reliability, criterion validity, and construct validity [18–21]. An optimal clinical cut-off point for a generalized anxiety disorder diagnosis is estimated to be a score of 10 or greater [18], and detecting anxiety disorders in general is estimated to be a score of 8 or greater [22]. The GAD-7 is also a reliable and valid measure of anxiety symptom severity [20,21].

The Icelandic version of the GAD-7 demonstrates good reliability and validity. Internal consistency is high in both non-clinical (α = .86–.88) [23,24] and clinical samples (α = .87) [23,25], with acceptable test–retest reliability in non-clinical participants (r = .58) [23]. Convergent and divergent validity are supported by strong correlations with related measures and weaker associations with unrelated measures [23,24]. Construct validity is also indicated by a one-factor structure [25] and appropriate item response patterns [24]. Criterion validity is adequate, with sensitivity (66.7%) and specificity (78.2%) at the optimal cut-off point of  ≥ 7 [23]. Sensitivity decreases and specificity increases at higher thresholds (≥ 8: 59.9%/83.3%; ≥ 10: 51.0%/88.5%) [23], aligning with findings that ≥ 10 identifies about 40% and ≥ 8 about 51% of participants as at risk [25]. Drawing on findings from the Icelandic validation study and research on cut-off points for detecting anxiety disorders more generally, the threshold was set at ≥ 8.

#### The Patient Health Questionnaire (PHQ-9).

The PHQ-9 [26] is a nineitem self-report questionnaire used to assess the presence and frequency of depressive symptoms in the past two weeks with good internal consistency and test-retest reliability, as well as  good criterion and construct validity [27,28]. Large-scale evaluations estimate that an optimal clinical cut off point for a major depressive disorder diagnosis is 10 or greater [27], though recent data-driven approaches suggest a lower threshold of 8 or greater [28].

The Icelandic version of the PHQ-9 demonstrates adequate to good reliability and validity. Internal consistency is adequate in non-clinical samples (α = .66–.84) [29,30] and good in clinical samples (α = .84–.87) [30,31]. Convergent and divergent validity is supported by stronger correlations with related measures and weaker associations with unrelated measures [29,30]. In addition, higher scores on the PHQ-9 predicted a greater likelihood of being diagnosed with major depressive disorder on the MINI [30]. The optimal cut-off for detecting major depressive disorder on the Icelandic version of the PHQ-9 is estimated at ≥ 12 [31], while a subsequent clinical study reported a similar optimal threshold of ≥ 11 (AUC = .83; sensitivity = 73%, specificity = 81%) [32]. Based on Icelandic validation studies indicating optimal cut-offs between 11 and 12 [31,32], a threshold of ≥ 12 was applied to define probable depressive disorders in this sample.

#### The Satisfaction with Life Scale (SWLS).

The SWLS [33] is a five item self-report measure to assess global life satisfaction that has good internal consistency, test-retest reliability, and construct validity [33–36]. Scores within 5–9 indicate extreme dissatisfaction, 10–14 dissatisfaction, 15–19 slight dissatisfaction, 21–25 slight satisfaction, 26–30 satisfaction, and 31–35 extreme satisfaction with life [33].

The Icelandic version of the SWLS has shown satisfactory psychometric properties. Internal consistency is adequate to good in non-clinical samples (α = .79–.88) [37,38], and test–retest reliability among university students is high (r = .88) [38]. Convergent and divergent validity are supported by stronger correlations with related constructs and weaker associations with unrelated constructs [37,38], and the scale demonstrates good discriminant validity with well-functioning items and response options [38]. Construct validity is further supported by a one-factor solution [37].

### Demographics

Information about recorded sex and age was received from the Registers Iceland. Other demographics information was collected from participants via self-report measures.

#### The Icelandic Registry of Primary Health Care Contacts and the Icelandic Hospital Discharge Registry.

The registries [39] contain information on primary care visits, outpatient hospital visits (including emergency room visits), and inpatient admissions, along with International Classification of Diseases (ICD) codes given by the treating physician. Psychiatric disorder codes included in the current study ranged from F00−F99. Inpatient data are available from 1999, primary care data from 2004, and hospital outpatient data from 2010, all through 2019. Registration of chronic disease diagnoses in Icelandic healthcare registries have recently been shown to have >90% validity for most diseases, however, psychiatric diagnoses were not assessed [40].

#### The Icelandic Prescription Medicines Registry.

The Icelandic Prescription Medicines Registry [41] contains information on the Anatomical Therapeutic Chemical (ATC) codes for prescription medication. Psychotropic medication codes included in the current study are N05A (antipsychotics), N05B (anxiolytics), N05C (hypnotics and sedatives), N06A (antidepressants), N06B (psychostimulants and nootropics), and R06AD02 (antihistamines for systemic use). Available data covered the years from 2002 until July 2019.

### Ethics statements

Approval for the study was received from the Icelandic National Bioethics Committee (Number 16 − 022, date: 2016 − 04 − 26) and the Icelandic Data Protection Agency. The Icelandic Directorate of Health approved access to national healthcare registries. The project was pre-registered on October 31, 2017 (trial registration number: NCT03327597). Participants were able to provide informed consent electronically or by a written consent form sent by mail.

### Procedure

Participants could register for the study either electronically or by mail. Participants who registered electronically were asked to complete self-report measures assessing psychiatric health between September 15^th^, 2016, and February 20^th^, 2018. Data from medical registries on psychiatric diagnoses and filled psychotropic medication prescriptions (diagnoses and filled prescriptions in the past 12 months were assessed one year from registration to the study, i.e., September 15^th^, 2015, and February 20^th^, 2017) were linked with self-report measures. Information on sex and age received from the Registers Iceland was also linked to data from medical registries and self-report measures. Participants who registered (electronically or by mail) and provided email addresses were able to provide additional demographic information.

### Statistical analyses

The percentage of participants with a psychiatric diagnosis in the year prior to registering for the study was calculated, as well as the percentage with diagnoses in the preceding 20 years (i.e., 1999–2019). The percentage of participants who filled a psychotropic prescription in the year prior to registering for the study was calculated, as well as the percentage of participants that filled prescriptions in the preceding 17 years (i.e., 2002–2019). The total number of filled benzodiazepinerelated drug or benzodiazepine derivative prescriptions was calculated over a one-year period (between February 20^th^, 2016, and February 20^th^, 2017). In addition, the percentage of individuals with one, two, or more than two filled prescriptions was calculated to quantify the extent of repeated or multi-agent use. Cumulative benzodiazepine exposure was quantified by summing all dispensed DDDs per individual and summarizing distributions using both broad (0–99, 100–499, 500–999, 1,000–4,999) and 15-DDD intervals. Descriptive statistics (mean, median, IQR) were used to characterize overall exposure and per-prescription dosing. Logistic regression (providing odds ratios [OR]) was conducted in two separate analyses to test whether sex and age were associated with having a psychiatric diagnosis and having filled a psychotropic prescription in the preceding year. The model fit criteria, or the AIC (Akaike Information Criterion), and significance test results for main and interaction effects between models were used to select an appropriate model. 95% confidence intervals for the ORs were calculated. Chi-square tests (χ^2^; providing the point percentage difference between the two groups) were conducted to compare the percentage of participants who filled a psychotropic prescription in the preceding year and across the entire registry data period (i.e., the preceding 1–17 years) with and without a psychiatric diagnosis in the preceding year and across the entire registry data period (i.e., the preceding 1–20 years). In addition, a 95% confidence interval for the percentage difference was calculated.

Descriptive statistical analyses for anxiety and depressive symptoms, and life satisfaction were conducted. Multiple regression analyses (providing regression coefficients) were conducted to test whether sex and age were associated with anxiety and depressive symptoms, or satisfaction with life. In addition, multiple regression analysis was conducted to assess whether self-reported anxiety and depressive symptoms, along with psychiatric disorder status, were associated with self-reported life satisfaction. Optimal transformations for the dependent variables were identified using Box-Cox and Yeo-Johnson transformations, along with normal and residual plots to address non-normality, non-linearity, heteroscedasticity, and outliers. Inverse transformation was used for GAD-7 (1 / (GAD7+1)), square root transformation for PHQ-9 (PHQ), and squared transformation for SWLS (SWLS^2). The model fit criteria, or the AIC, visual inspection of graphs, and significance test results for main and interaction effects between models were used to select an appropriate model. 95% confidence intervals were calculated. To compare means on self-report measures between individuals with and without a psychiatric diagnosis (and separately compare means between those with an anxiety or a depressive disorder), and individuals who filled or did not fill psychotropic prescriptions in the preceding year, independent *t*-tests (providing the mean difference) were conducted, and 95% confidence intervals were calculated. A Levene’s test was conducted to tes*t* the homogeneity of variances. All statistical tests were two-tailed, and statistical significance was set at the 0.05 alpha level. RStudio versions 1.3.1073 to 2025.09.1 + 401 were used for all statistical analyses [42].

## Results

### Reported data

Psychiatric diagnoses and filled psychotropic prescriptions data were available for all participants who registered for the study. Out of the 80,733 participants, 59,832 (74.1%) registered electronically (while 20,896 [25.9%] registered by mail) and were asked to respond to questionnaires at that time. Of those, 61.3% (36,674) completed the GAD-7, 61.6% (36,838) completed the PHQ-9, and 62.2% (37,215) completed the SWLS. Compared to responders, non-responders were more likely to be male, older, less educated, retired, and have lower salaries (See Table in S1 Table). A questionnaire assessing demographics was sent out to participants who provided an email address (72,902; 90.3%). Of those between, 28,039 and 42,395 (38.5–58.2%) completed the demographic questions. Missing data were excluded from analysis (i.e., no statistical computations were conducted to handle missing data). Age and sex were available from Registers Iceland for all participants.

### Participant demographics

The current study sample included a total of 80,733 participants with a mean age of 60.0 years (*SD* = 11.7; range: 40–104), and thereof, 43,656 were females (54.1*%*; Table 1).

**Table 1:** Participant demographics.

Characteristic	*M (SD)* ^a^ *; Frequency (%)*
Age^b^	60.0 (11.7)
Sex^b^	
Female	43,656 (54.1%)
Male	37,077 (45.9%)
Birthplace^c^	
Iceland	27,258 (97.2%)
Other	781 (2.8%)
Housing^c^	
Homeowner, renting or living with partner	31,432 (97.3%)
Other	856 (2.7%)
Education^c^	
Some formal education	834 (2.1%)
Lower-secondary school	8,071 (20.3%)
Upper-secondary school	8,244 (20.8%)
Vocational college degree	8,047 (20.3%)
University education	14,482 (36.5%)
Employment status^c,d^	
Employed part or full-time	27,070 (63.9%)
Unemployed	393 (0.9%)
Maternity leave	20 (0.05%)
Student	631 (1.5%)
Homemaker	929 (2.2%)
Sick leave	756 (1.8%)
Disability	3,292 (7.8%)
Retired	10,904 (25.7%)
Salary^c,e^	
Less than 1,110	649 (1.7%)
1,111–2,220	7,108 (18.4%)
2,221–3,700	11,447 (29.6%)
3,701–5,190	9,166 (23.7%)
5,191–6,670	5,195 (13.4%)
More than 6,671	5,168 (13.3%)

The table presents descriptive characteristics of study participants, including mean age with standard deviation (SD) and frequency distributions of sex, birthplace, housing status, education level, employment status, and monthly salary. Percentages are based on available data for each variable.

^a^*M* = mean of total scores; *SD* = standard deviation of total scores; *n* = number of participants.

^b^Information for age and sex was obtained from public registries and available for all participants providing informed consent.

^c^Information on birthplace, housing, education, employment status, and salary is only available for a part of the sample.

^d^Not mutually exclusive categories.

^e^Salary per month before taxes (total income) was converted from the Icelandic krona to Euros based on currency exchange rates on the 12th of April 2019, which is the median date of data collection.

### Psychiatric diagnoses

**Fig 1:**
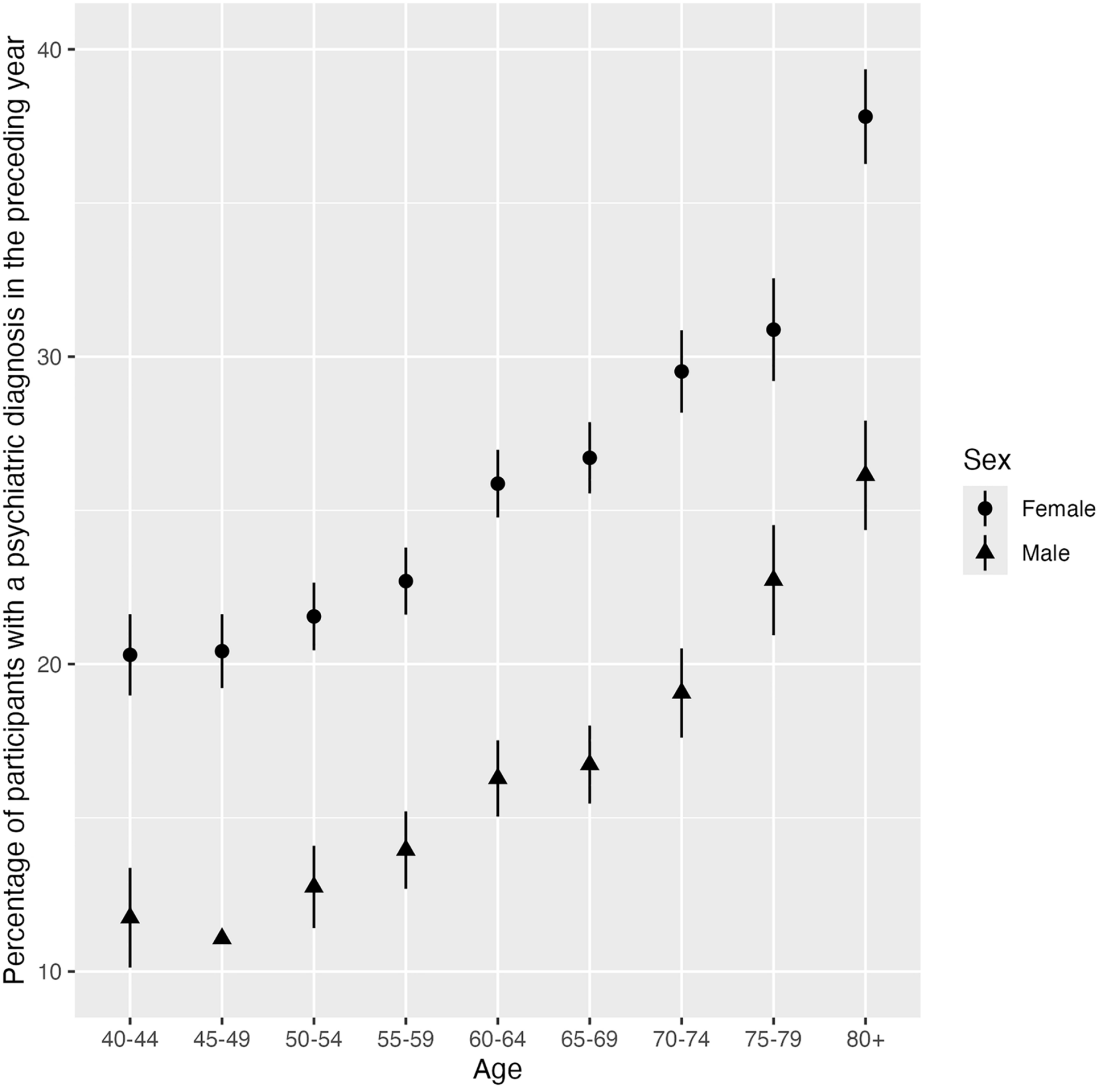
Percentages of individuals with a psychiatric diagnosis in the preceding year by age group and sex (with the 95% confidence intervals based on standard errors for each age group by sex). The figure shows the percentage of individuals with at least one psychiatric disorder diagnosis across age groups, stratified by sex. Prevalence increased with age and was consistently higher among females than males. Error bars indicate 95% confidence intervals.

Of the total sample, 16,764 individuals (20.8%) received one or more psychiatric diagnoses in the preceding year, and 36,227 (44.9%) in the preceding 20 years. Logistic regression analyses revealed that males had a lower probability of receiving a diagnosis in the preceding year (5,885 [15.9%]; OR 0.40, 95% CI 0.33–0.48; p < 0.0001) compared to females (10,879 [24.9%]; Table 2 and Table in S2 Table). In addition, the odds of being diagnosed with a psychiatric disorder increased with each 10 years of participants´ age (OR 1.22 95% CI 1.20–1.25; p < 0.0001) with the diagnostic rate for the oldest age group (80 years and older; 1,321 [32.3%]) being double that of the youngest age group (40–44 years old; 1,606 [16.7%]). The interaction effect between sex and 10 years of age was statistically significant (OR 1.05, 95% CI 1.02–1.09; p < 0.001), with a stronger age gradient for females. The sex difference ranged from 8.6 percentage points in the youngest age group to 11.7 percentage points the oldest age group. For further details on sex breakdowns across age groups, see Fig 1. Rates of anxiety and mood disorders also differed (ranging between 8.1–10.1%) based on age group (i.e., 40–44: 10.1%, 45–49: 9.5%, 50–54: 9.4%, 55–59: 8.9%, 60–64: 9.2%, 65–69: 8.1%, 70–74: 8.4%, 75–79: 9.4%, 80 + : 10.0%).

**Table 2:** Frequencies and percentages of individuals with a psychiatric diagnosis within the preceding year.

	Psychiatric diagnosis		
Categories of disorders along with ICD codes^a^	Independent of sex	Males	Females
	*n (%)* ^b^	*n (%)* ^b^	*n (%)* ^b^
Organic, including symptomatic, mental disorders (F00 − F09)	470 (0.58%)	219 (0.59%)	251 (0.57%)
Mental and behavioral disorders due to psychoactive substance use (F10 − F19)	1,157 (1.43%)	589 (1.59%)	568 (1.30%)
Schizophrenia, schizotypal, and delusional disorders (F20 − F29)	120 (0.15%)	59 (0.16%)	61 (0.14%)
Mood [affective] disorders (F30 − F39)	4,320 (5.35%)	1,377 (3.71%)	2,943 (6.74%)
Unipolar disorders (F33 − F33.9, F34.1)	859 (1.06%)	241 (0.65%)	618 (1.42%)
Depressive episode (F32 − F32.9)^c^	3,329 (4.12%)	1,075 (2.90%)	2,254 (5.16%)
Bipolar disorders (F30 − F31.9, F34.0)	294 (0.36%)	110 (0.30%)	184 (0.42%)
Phobic and other anxiety disorders (F40 − F41.9)	4,098 (5.08%)	1,078 (2.91%)	3,020 (6.92%)
Obsessive-compulsive disorder (F42 − F42.9)	59 (0.07%)	21 (0.06%)	38 (0.09%)
Reaction to severe stress and adjustment disorders (F43 − F43.9)	1,465 (1.82%)	350 (0.94%)	1,115 (2.55%)
Dissociative [conversion] disorders (F44 − F44.9)	14 (0.02%)	3 (0.01%)	11 (0.03%)
Somatoform disorders (F45 − F45.9)	45 (0.06%)	18 (0.05%)	27 (0.06%)
Eating disorders (F50 − F50.9)	19 (0.02%)	0 (0.00%)	19 (0.04%)
Nonorganic sleep disorders (F51 − F51.9)	9,171 (11.36%)	2,990 (8.06%)	6,181 (14.16%)
Sexual dysfunction, not caused by organic disorder or disease (F52 − F52.9)	557 (0.69%)	552 (1.49%)	5 (0.01%)
Disorders of adult personality and behavior (F60 − F69)	113 (0.14%)	53 (0.14%)	60 (0.14%)
Mental retardation (F70 − F79)	53 (0.07%)	19 (0.05%)	34 (0.08%)
Disorders of psychological developments (F80 − F89)	23 (0.03%)	11 (0.03%)	12 (0.03%)
Behavioral and emotional disorders with onset usually occurring in childhood and adolescence (F90 − F98)	365 (0.45%)	151 (0.41%)	214 (0.49%)
Total^d^	16,764 (20.77%)	5,885 (15.87%)	10,879 (24.92%)

The table presents the number and percentage of participants with the psychiatric disorder diagnosis in the year preceding study enrollment, categorized by ICD-10 diagnostic groups (F00–F99). Numbers are shown for the total sample and stratified by sex. Percentages are based on the total number of study participants (*n* = 80,733).

^a^ICD = International Classification of Diseases version 10.

^b^*n* = number of participants.

^c^Depressive episode is not included in unipolar disorders because it is unclear whether the episode occurred in unipolar or bipolar disorder.

^d^Numbers are based on those with at least one psychiatric diagnosis in the past year.

Nonorganic sleep disorders (F51; 9,171 [11.4%]) were the most common diagnosis, followed by other anxiety disorders (F41; 3,964 [4.9%]) and a depressive episode (F32; 3,329 [4.1%]). Fewer males received a diagnosis in the previously mentioned categories compared to females (F51; 2,990 [8.1%] versus 6,181 [14.2%]; F41; 1,033 [2.8%] versus 2,931 [6.7%]; F32; 1,075 [2.9%] versus 2,254 [5.2%]). Of the total sample, 6.5% (5,282) were diagnosed with at least one anxiety disorder (i.e., phobic anxiety disorders [F40–F40.9], other anxiety disorders [F41–F41.9], obsessive-compulsive disorder [F42–F42.9], or reaction to severe stress and adjustment disorders [F43–F43.9]) and 5.0% (4,060) with a depressive episode (F32–F32.9) or depressive disorder (i.e., recurrent depressive disorder [F33–F33.9] and dysthymia [F34.1]), or both. About half of those who received at least one diagnosis (9,385 [11.6%]) had one or more of the following: a substance use disorder, a psychotic disorder, a mood disorder, an anxiety disorder, a somatoform disorder, or an eating disorder diagnosis (Table 2 and Table in S2 Table).

### Psychotropic medications

**Fig 2:**
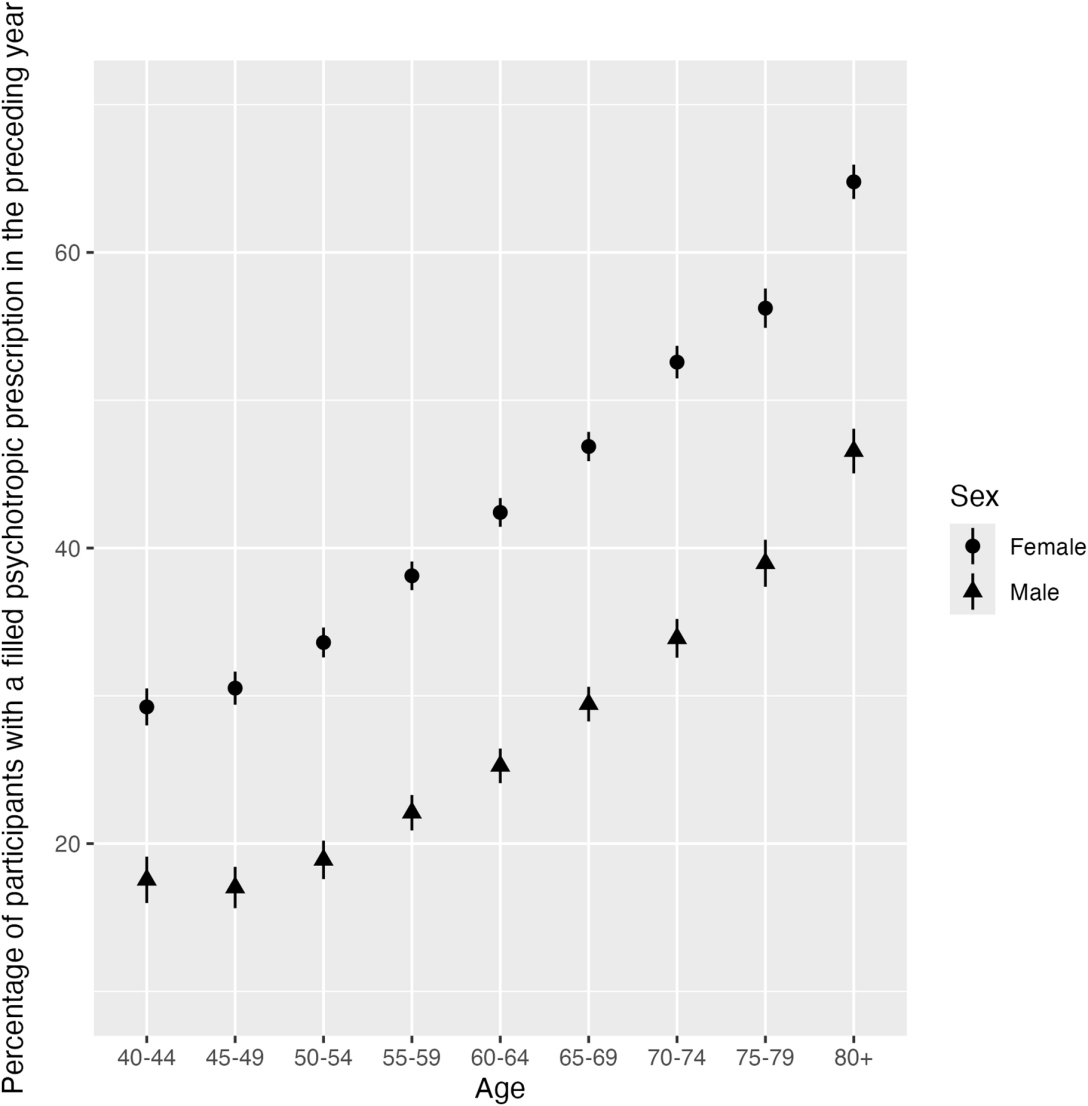
Percentages of filled psychotropic prescriptions in the preceding year by age group and sex (with the 95% confidence intervals based on standard errors for each age group by sex. The figure shows the percentage of individuals with at least one filled psychotropic prescription in the preceding year across age groups, stratified by sex. Prescription rates increased with age and were consistently higher among females than males. Error bars indicate 95% confidence intervals.

Of the total sample, 27,642 (34.2%) filled at least one psychotropic medication in the 12 months preceding registration for the study, and 48,347 (59.9%) in the preceding 17 years. Logistic regression revealed that males had a lower probability of filling prescriptions in the preceding year (9,660 [26.1%]; OR 0.45, 95% CI 0.38–0.54; p < 0.0001) compared to females (17,982 [41.2%]; Table 3 and Table in S3 Table). In addition, the odds of filling a prescription increased with each 10 years of participants´ age (OR 1.44, 95% CI 1.41–1.47; p < 0.0001) with the filled prescription rate for the oldest age group (80 years and older; 2,793 [56.2%]) being more than double that of the youngest age group (40–44 years old; 1,924 [24.3%]). The interaction effect between sex and 10 years of age was not statistically significant (OR 1.01, 95% CI 0.98–1.03; p = 0.646), however, the sex difference ranged from 11.7 percentage points in the youngest age group to 18.2 percentage points in the oldest age group. For further details on sex breakdowns across age groups, see Fig 2.

**Table 3:** Frequencies and percentages of individuals using psychotropic medications within the preceding year.

ATC code^a^	Independent of sex	Males	Females
	*n (%)* ^b^	*n (%)* ^b^	*n (%)* ^b^
Antipsychotics (N05A)	3,151 (3.90%)	1,125 (3.03%)	2,026 (4.64%)
Anxiolytics (N05B)	9,165 (11.35%)	2,820 (7.61%)	6,345 (14.53%)
Hypnotics and sedatives (N05C)	15,584 (19.30%)	5,410 (14.59%)	10,174 (23.31%)
Antidepressants (N06A)	14,109 (17.48%)	4,483 (12.09%)	9,626 (22.05%)
Psychostimulants and nootropics (N06B)	811 (1.01%)	355 (0.96%)	456 (1.04%)
Antihistamines for systemic use (Phenergan; R06AD02)	1,508 (1.87%)	490 (1.32%)	1,018 (2.33%)
Total^c^	27,642 (34.24%)	9,660 (26.05%)	17,982 (41.19%)

The table presents the number and percentage of participants who received a prescription for psychotropic medications within the year preceding study enrollment, categorized according to the Anatomical Therapeutic Chemical (ATC) classification system. Numbers are shown for the total sample and stratified by sex. Percentages are based on the total number of study participants (*n* = 80,733).

^a^ATC = The Anatomical Therapeutic Chemical (ATC) Classification System.

^b^*n* = number of participants.

Overall, 19,639 (24.3%) individuals filled one or more prescriptions for benzodiazepine related drugs (N05CF; the most commonly filled psychotropic prescription; 14,697 [18.2%]), or benzodiazepine derivatives (N05BA; the third most filled psychotropic prescription; 8,514 [10.6%]; N05CD; 815 [1.01%]), possibly from more than one category, in the preceding year. Between February 20^th^, 2016, and February 20^th^, 2017, a total of 167,044 prescriptions for benzodiazepine-related drugs or benzodiazepine derivatives were filled by 20,452 individuals. Most individuals filled prescriptions for either one (15,456; 75.57%) or two (4,258; 20.82%) distinct ATC codes in these drug classes. Benzodiazepine derivatives accounted for 44,576 prescriptions across 8,935 individuals (N05BA) and 7,120 prescriptions across 828 individuals (N05CD). Benzodiazepine related drugs (N05CF) accounted for 115,348 prescriptions across 15,262 individuals. Among all 20,452 individuals, 4.720 (23.08%) filled one, 2.202 (10.77%) two, 1.387 (6.78%) three, 1.172 (5.73%) four, 946 (4.63%) five, and 10.025 (49.02%) more than five prescriptions during the one-year period. General practitioners accounted for the majority of prescriptions (86,380; 51.71%), followed by physicians with a general medical license (27,390; 16.40%), trainees/residents (10,663; 6.38%), and psychiatrists (9,615; 5.76%), while all other specialties combined contributed 32,996 prescriptions (19.75%). Cumulative DDD exposure varied markedly across individuals, with the highest concentration in the 30–44 DDD range (3,125; 15.28%), followed by substantial proportions in the 0–14 DDD (1,811; 8.85%), 60–74 DDD (1,471; 7.19%), and 90–104 DDD (1,060; 5.18%) intervals. Overall, 8,929 (43.66%) of individuals received fewer than 100 DDDs during the year, 9,572 (46.80%) received 100–499 DDDs, and only a small minority received 500 or more DDDs (1,602; 7.83% in the 500–999 range; 349; 1.71% in the 1,000–4,999 range). Mean cumulative exposure was 217,97 DDDs (median 141.60; IQR 30.00–332.00), with a maximum of 4,260 DDDs. At the prescription level, individuals received an average of 26.69 DDDs per fill (median 30.00), indicating relatively consistent dosing across prescriptions. Excluding individuals diagnosed with nonorganic sleep disorders (F51–51.9), 11,625 of 71,562 (16.3%) participants filled one or more prescriptions for benzodiazepine-related drugs (N05CF) or benzodiazepine derivatives (N05BA; N05CD), possibly from more than one category, in the preceding year. The second most common class of psychotropic prescriptions that were filled was selective serotonin reuptake inhibitors (SSRIs; N06AB; 9,224 [11.4%]; Table 3 and Table in S3 Table). Fewer males (N05CF; 5,090 [13.7%]; N06AB; 2,911 [7.9%]; N05BA; 2,602 [7.0%]) filled prescriptions in the previously mentioned categories compared to females (N05CF; 9,607 [22.0%]; N06AB; 6,313 [14.5%]; N05BA; 5, 912[13.5%]). Additionally, the percentage of filling a benzodiazepine prescription increased steadily with participants´ age (ranging from 12.4–48.3% across age groups).

The results from a χ^2^ test revealed that individuals with a psychiatric diagnosis in the preceding year were more likely to obtain psychotropic medications (14,943 [89.1%]) compared to individuals without a psychiatric diagnosis in the preceding year (12,699 [19.9%]; point percentage difference 69.2%, 95% CI 68.7–69.9; p < 0.0001). Individuals diagnosed with a psychiatric disorder over the preceding 20 years were more likely to have filled a psychotropic prescription in the preceding 17 years (33,065 [91.3%]) compared to individuals without a psychiatric diagnosis (15,282 [34.3%]; point percentage difference 57.0%, 95% CI 56.4–57.5; p < 0.0001).

### Psychiatric symptoms and severity

In the current sample, the GAD-7 (Cronbach’s α = 0.88), PHQ-9 (Cronbach’s α = 0.84), and SWLS (Cronbach’s α = 0.88) had good internal consistency. Of the 36,674 participants who completed the GAD-7, most participants reported no or mild anxiety symptoms (34,576 [94.3%]), however, 2,098 (9.5%) met the clinical cut-point for an anxiety disorder. The results from multiple regression (*F*[2, 36,671]=519.60, p < 0.0001, *R*^*2*^ = 0.03) revealed that females reported higher anxiety symptoms (*b* −0.06, 95% CI −0.05– −0.06; p < 0.0001), and symptoms were lower in older participants (*b* 0.01, 95% CI 0.00–0.01; p < 0.0001; Table 4). Of the 36,838 participants who completed the PHQ-9, most participants reported minimal or mild depressive symptoms (32,697 [88.8%]), but 2,604 [7.1%] met the clinical cut-point for major depressive disorder. Overall (*F*[2, 36,835]=417.8,0, p < 0.0001, *R*^*2*^ = 0.02), females reported higher depressive symptoms (*b* 0.23, 95% CI 0.21–0.25; p < 0.0001), and symptoms were lower in older participants (*b* −0.01, 95% CI −0.01– −0.01; p < 0.0001; Table 4). Of the 37,215 participants who completed the SWLS, most (33,183 [89.2%]) participants reported being slightly satisfied (11,101 [29.8%]), satisfied (16,368 [44.0%]), or extremely satisfied with life (5,714 [15.4%]). Additionally (*F*[2, 37,212]=36.69, p < 0.0001, *R*^*2*^ = 0.002), females reported higher satisfaction with life (*b* 17.65, 95% CI 12.95–22.35; p < 0.0001), and satisfaction was higher for older participants (*b* 0.68, 95% CI 0.44–0.92; p < 0.0001; Table 4).

**Table 4:** Descriptive statistics for depressive and anxiety symptoms, and satisfaction with life by sex and age group.

	Self-report measurement					
	GAD-7^a^		PHQ-9^a^		SWLS^a^	
Age group	Males	Females	Males	Females	Males	Females
	*M (SD)* ^b^ *n*	*M (SD)* ^b^ *n*	*M (SD)* ^b^ *n*	*M (SD)* ^b^ *n*	*M (SD)* ^b^ *n*	*M (SD)* ^b^ *n*
40–44	3.8 (3.6) 1,969	4.3 (3.7) 3,036	4.6 (4.1) 1,975	5.3 (4.3) 3,050	25.8 (4.6) 2,001	26.5 (4.6) 3,078
45–49	3.5 (3.4) 2,395	3.9 (3.6) 3,557	4.5 (4.0) 2,406	5.1 (4.3) 3,570	25.6 (4.6) 2,435	26.4 (4.7) 3,607
50–54	3.3 (3.4) 2,594	3.6 (3.6) 3,952	4.3 (4.1) 2,602	5.0 (4.5) 3,968	25.6 (4.8) 2,619	26.2 (4.8) 4,003
55–59	3.0 (3.2) 2,665	3.6 (3.6) 3,618	4.1 (4.1) 2,674	5.0 (4.3) 3,635	25.6 (4.8) 2,703	25.9 (4.8) 3,667
60–64	2.8 (3.2) 2,456	3.4 (3.4) 2,927	3.9 (3.9) 2,470	4.8 (4.2) 2,942	26.0 (4.5) 2,489	26.1 (4.6) 2,978
65–69	2.4 (2.9) 1,939	3.0 (3.2) 2,105	3.5 (3.7) 1,944	4.3 (4.0) 2,120	26.4 (4.4) 1,963	26.6 (4.5) 2,137
70–74	2.2 (2.8) 1,127	3.1 (3.1) 1,021	3.2 (3.5) 1,131	4.2 (3.6) 1,031	26.8 (4.2) 1,139	26.5 (4.1) 1,048
75–79	2.2 (2.7) 486	2.9 (3.2) 380	3.5 (3.5) 490	4.4 (4.1) 381	27.2 (4.3) 497	26.2 (4.3) 387
80+	2.1 (2.8) 238	2.7 (3.0) 209	3.8 (3.9) 240	4.8 (4.1) 209	27.3 (3.9) 251	26.5 (4.1) 213
Total	3.0 (3.3) 15,869	3.6 (3.5) 20,805	4.1 (4.0) 15,932	4.9 (4.3) 20,906	26.0 (4.6) 16,097	26.3 (4.6) 21,118

The table presents mean scores (M) and standard deviations (SD) for the GAD-7, PHQ-9, and SWLS across age groups, stratified by sex. Higher scores on the GAD-7 and PHQ-9 indicate greater symptom severity, whereas higher SWLS scores indicate greater life satisfaction.

^a^GAD-7 = The Generalized Anxiety Disorder Scale; PHQ-9 = The Patient Health Questionnaire; SWLS = Satisfaction with Life Scale.

^b^*M* = mean of total scores; *SD* = standard deviation of total scores. *n* = number of participants.

The multiple regression analysis (*F*[3, 36,670]=5,063, p < 0.0001, *R*^*2*^ = 0.29) revealed that higher levels of self-reported anxiety (*b* −82.02, 95% CI −93.31– −70.74; p < 0.0001) and depressive symptoms (*b* −138.83, 95% CI −142.19– −135.47; p < 0.0001), along with psychiatric disorder status (*b* −19.78, 95% CI −24.99– −14.56; p < 0.0001) were associated with lower self-reported satisfaction with life. Additionally, an interaction effect between anxiety and depressive symptoms was significant (*b* 81.15, 95% CI 74.94–87.35; p < 0.0001). Among individuals with lower levels of depressive symptoms, increases in anxiety symptoms are associated with decreases in satisfaction with life. However, as depressive symptoms increase, the negative impact of anxiety symptoms on satisfaction with life becomes less pronounced.

Individuals diagnosed with a psychiatric disorder in the preceding year or who filled a prescription for psychotropic medication in the preceding year, including those meeting both criteria, reported higher anxiety symptoms (2.04 and 1.94 points higher, 95% CI 1.94–2.15 and 95% CI 1.86–2.03; *t*[8,740.5] = 37.66 and *t*[16,532] = 45.55, p < 0.0001, respectively), higher depressive symptoms (2.97 and 2.89 points higher, 95% CI 2.85–3.10 and 95% CI 2.79–2.99; *t*[8,738] = 45.72 and *t*[16,259] = 56.64, p < 0.0001, respec*t*ively), and lower satisfaction with life (2.22 and 2.03 points lower, 95% CI 2.09–2.35 and 95% CI 1.92–2.14; *t*[9,466.8] = 32.78 and *t*[18,617] = 36.88, p < 0.0001, respec*t*ively; Table 5). Individuals diagnosed wi*t*h an anxiety disorder in the preceding year reported higher anxiety symptoms (2.85 points higher, 95% CI 2.67–3.04; *t*[2,624.8] = 30.35, p < 0.0001), and individuals diagnosed with a depressive episode or depressive disorder in the preceding year, or bo*t*h, report higher depressive symptoms (4.43 points higher, 95% CI 4.17–4.69; *t*[2,016] = 33.48, p < 0.0001).

**Table 5:** Comparisons of anxiety and depressive symptoms, and satisfaction with life of individuals with or without a psychiatric diagnosis and filled psychotropic prescriptions in the preceding year.

	Psychiatric diagnosis			Filled psychotropic medication prescriptions		
	Without *M (SD)*^b^ *n*	With *M (SD)*^b^ *n*	*t-value*	Without *M (SD)*^b^ *n*	With *M (SD)*^b^ *n*	*t-value*
GAD-7^a^	3.0 (3.1) 29,711	5.0 (4.3) 6,963	37.7***	2.8 (2.9) 25,393	4.7 (4.1) 11,281	45.6***
PHQ-9^a^	4.0 (3.7) 29,834	6.9 (5.1) 7,004	45.7***	3.6 (3.4) 25,498	6.5 (4.9) 11,340	56.6***
SWLS^a^	26.6 (4.4) 30,125	24.3 (5.3) 7,090	32.8***	26.8 (4.2) 25,724	24.7 (5.2) 11,491	36.9***

The table presents mean scores and standard deviations (SD) for the GAD-7, PHQ-9, and SWLS, comparing individuals with and without a recorded psychiatric diagnosis and with and without filled psychotropic medication prescriptions. Higher GAD-7 and PHQ-9 scores indicate greater symptom severity, while higher SWLS scores indicate greater life satisfaction.

^a^GAD-7 = The Generalized Anxiety Disorder Scale; PHQ-9 = The Patient Health Questionnaire; SWLS = Satisfaction with Life Scale.

^b^*M* = mean of total scores; *SD* = standard deviation of total scores. *n* = number of participants. *t-value* = independent samples t-tests comparing the groups.

***p < 0.0001.

## Discussion

In this large nationwide study of 80,733 participants, a fifth of the population received one or more psychiatric diagnoses within the preceding year and nearly half within the past 20 years. These findings reveal the widespread prevalence of psychiatric disorders across the population and underscore the substantial demand for psychiatric health services. However, diagnoses are non-specific and broad in nature, which limits the ability to provide evidence-based psychiatric services. Filled psychotropic medication prescriptions were also common, with a third of the population filling at least one psychotropic medication prescription within the preceding year (20% without a recorded psychiatric diagnosis) and more than half filling at least one prescription over the past 17 years. Such extensive use, especially among individuals without a recorded psychiatric diagnosis, suggests that medications are frequently used to manage distress or symptoms outside of clearly defined diagnostic criteria. Benzodiazepine prescriptions were filled by a quarter of the population in the preceding year, although clinical practice guidelines worldwide discourage benzodiazepines as a first-line treatment [43]. Our findings highlight widespread filling of psychiatric medication prescriptions, including among individuals without a diagnosis, and raise concerns about prescribing practices, particularly regarding benzodiazepines.

The prevalence of psychiatric diagnoses (21%) in the current study is substantially higher than both global [1,2] and Icelandic IHME estimates (13%; excluding substance use disorders) [44,45]. However, the findings are consistent with the results of a previous study from the Icelandic capital area, in which 20% of individuals aged 34−76 years old met criteria for a disorder (including substance use disorders; based on a diagnostic interview) [46], as well as with 2024 US survey data reporting a comparable prevalence of 23% among individuals aged 18 years or older (excluding substance use disorders; survey classifications were statistically modeled using data from previous clinical interviews) [47]. The IHME estimates are generated through statistical modeling that integrates data from population-based surveys, epidemiological studies, and, where available, healthcare registry data [48]. In contrast, the current study data are based on healthcare registry data reflecting clinically recorded diagnoses within Iceland’s national healthcare system, likely providing a more accurate depiction of the burden of psychiatric disorders in the population. As the present study encompasses a single, well-defined nationwide population and includes data from primary care, outpatient (including emergency room), and inpatient settings, the registry-based data likely capture help-seeking behavior for psychiatric problems, including among individuals who do not meet formal diagnostic criteria. This approach, therefore, offers a realistic estimate of the resulting burden on the healthcare system. Overall, the results indicate that psychiatric problems are widespread and underscore the need for accessible, evidence-based psychiatric care across the population.

The most common psychiatric diagnoses were nonorganic sleep disorders (including insomnia, hypersomnia, and sleep terrors), followed by other anxiety disorders (including panic disorder and generalized anxiety disorder) and a depressive episode (including mild to severe depressive episodes with/without somatic syndrome and psychotic symptoms). This diagnostic pattern suggests that clinical practice often centers on prominent symptom presentations rather than clearly differentiated psychiatric disorders, possibly reflecting the lack of resources and practical constraints of general medical settings. The non-specific and broad nature of these diagnoses, which encompass a range of disorders with varying clinical presentations and treatment needs [43], provides limited guidance on appropriate care. For instance, a depressive episode diagnosis does not distinguish between conditions such as major depressive disorder, bipolar disorder, or schizoaffective disorder, making it difficult to determine the most appropriate course of treatment. Strengthening diagnostic accuracy through clinician training and the use of structured assessment tools could improve diagnostic accuracy, and therefore, the use of evidence-based treatment, ultimately enhancing patient outcomes. The findings of the current study somewhat align with the findings from a previous Icelandic study [46] in that the major categories of psychiatric diagnoses are similar, except for the lack of somatoform disorders and substance use disorders diagnoses in the current study. In the current study, the prevalence of those diagnosed with at least one anxiety disorder (i.e., F40–F43.9; 6.5%) or with a depressive episode (F32–F32.9) or depressive disorder (i.e., recurrent depressive disorder [F33–F33.9] and dysthymia [F34.1]; 5.0%), or both, was lower compared to an Icelandic study from 1991 (12% and 9%; respectively) [49] and higher but more similar compared to an Icelandic study from 2009 (6% and 3%; respectively) [46]. The higher prevalence in 1991 may partly reflect methodological and diagnostic differences, as earlier versions of the diagnostic system were more lenient in their criteria. These differences may reflect methodological variation, as earlier Icelandic studies were based on structured diagnostic interviews, whereas the current study is based on healthcare registry data. The two data sources may capture different aspects of psychiatric health: one reflecting the prevalence of psychiatric disorders meeting diagnostic criteria in the general population, and the other representing individuals seeking help and receiving diagnoses based on different assessment methods. Consequently, the observed variation may reflect shifts in awareness and help-seeking behavior regarding psychiatric problems that do not meet diagnostic criteria, as well as in diagnostic assessment practices.

Self-reported clinically severe anxiety and depressive symptoms corresponded closely to rates of anxiety disorders, and depressive episodes or disorders in the preceding year. The results suggest that physician-based diagnoses in routine healthcare settings may adequately capture most individuals experiencing clinically significant psychiatric symptoms. This correspondence indicates that diagnostic practices in Iceland are broadly aligned with symptom thresholds and reflect genuine mental health needs rather than diagnostic overreach. At the same time, some individuals who score above the threshold for anxiety disorders did not receive a diagnosis, possibly due to an under-recognition of some conditions such as post-traumatic stress disorder (PTSD). A prior study revealed similar prevalence rates of individuals in the general population who met the clinical cut-point on the GAD-7 [20] and PHQ-9 [50]. Thus, rates of clinically significant anxiety and depressive symptoms are consistent across studies and populations, and therefore, might be reflective of a broader pattern of psychiatric health. The consistency of these patterns across studies further supports the reliability of registry-based data for monitoring psychiatric health at the population level.

Females were equally or more likely than males to receive a diagnosis across all diagnostic categories and reported higher anxiety and depressive symptoms, indicating a greater psychiatric health burden among females. In the current study, females showed a higher psychiatric burden (25%) compared to males (16%), a pattern consistent with previous data from Europe indicating that psychiatric disorders are overall more prevalent among females (20%) than males (18%) [51]. The larger gender difference observed in this study may reflect differences in help-seeking behavior, with females more likely to seek psychiatric health support and, therefore, more likely to receive a diagnosis. This is consistent with females using more non-acute healthcare services than males across Europe [52].

Also, substance use disorder, which is more common among males, was not captured in the current study, which may further contribute to the observed gender gap [51]. Overall, the prevalence of psychiatric disorder diagnoses increased across age groups, with higher rates among older participants. However, anxiety and mood disorder followed a curvilinear pattern, with rates peaking in midlife, decreasing through older adulthood, and increasing again in the oldest age groups. Interestingly, older individuals reported lower levels of anxiety and depressive symptoms than younger individuals, despite having higher diagnostic rates. The discrepancy could be explained by greater healthcare contact among older individuals, providing more opportunities for psychiatric diagnoses and treatment. Lower self-reported symptoms may instead reflect generational attitudes toward psychiatric health or limitations of self-report measures in accurately capturing symptoms in older adults. Another possibility is that psychiatric disorders may be overdiagnosed in older age groups. Prior studies indicate lower rates of anxiety and mood disorders in older adults, including findings of low rates among individuals above 80 years old [53] and a steady decline across age groups [54]. The inconsistencies highlight the need to further clarify age-related diagnostic patterns to ensure that appropriate assessment methods and interventions are available for individuals in need.

Prevalence rates of filled psychotropic medication prescriptions were concerningly high, particularly among females and older adults, indicating that pharmacological treatment remains a predominant approach to managing psychiatric problems. The rates observed in the current study exceed those reported in a previous Icelandic study, in which 20% of individuals aged 17–75 years old reported using one or more psychotropic medications (i.e., antidepressants, tranquillizers, and sedatives) [55], and in a U.S. study showing a lower prevalence of 17% among adults aged 45 years and older [13]. These differences may reflect variations in study age ranges, healthcare access, medication adherence, and prescribing practices. Consistent with previous research, females and older adults were more likely to fill a prescription compared to males and younger adults [10,13]. Further investigation into the high rates among older females is warranted, as they may reflect both greater help-seeking behavior and a potential overreliance on pharmacological treatment in this group, possibly driven by an even more limited access to psychotherapy among older adults. Although filled psychotropic medication prescriptions were strongly associated with psychiatric diagnosis, a considerable number of individuals without a recorded psychiatric diagnosis also filled prescriptions. The overall high prevalence rates of filled psychotropic medication prescriptions may stem from physicians´ limited training in psychiatric assessment, time constraints in clinical practice, and a healthcare system that partly or fully reimburses medications while providing only partial coverage for psychotherapy, as well as lenient prescribing regulations. These findings highlight the need to reduce overreliance on psychotropic medication for psychiatric problems by strengthening diagnostic training, expanding access to reimbursed psychotherapeutic services, and implementing stricter regulations regarding prescribing practices. Regular review of long-term prescriptions, particularly among older adults, could promote safer and more appropriate treatment. Health policies that promote evidence-based treatment and enhance monitoring of prescribing patterns are essential to ensure effective psychiatric health care.

Filled benzodiazepine prescriptions were strikingly high in the current study and remained elevated even after excluding individuals with nonorganic sleep disorders, raising concerns about prescribing practices and treatment culture. International clinical guidelines discourage benzodiazepines as a first-line treatment for psychiatric disorders [43]. This recommendation is supported by evidence showing that long-term benzodiazepine use is associated with adverse outcomes, including falls [56], dementia [57], mortality [58], and misuse and dependence [59]. Furthermore, the IHME estimates that the global anxiety burden could be reduced by 71% with optimal treatment for anxiety disorders [60], and medications such as benzodiazepines are not considered an effective first-line treatment [43]. The reliance observed may reflect similar factors underlying the widespread use of psychotropic medications in general, including limited training in psychiatric assessment and limited access to psychotherapy. The rates of filled benzodiazepine prescriptions were much higher (24%) than reported use in the US in 2015–2016 (13%) and another European country in 2002 (9%) [59,61]. Among the Nordic countries, Iceland has the highest use of prescription benzodiazepine and benzodiazepine-like drugs [62,63], which may be explained by the limited availability of psychotherapy and less strict regulations on prescribing addictive medications compared to Denmark and Norway [62]. The prescribing patterns observed in this study indicate that less than one-fourth of individuals filled only a single benzodiazepine-related prescription, while the majority filled multiple prescriptions, with nearly half filling more than five prescriptions within one year. Cumulative DDD analyses further revealed substantial heterogeneity in exposure. Although most individuals received relatively low annual amounts, a clinically important subgroup accumulated high levels that clearly exceeded guidelines recommending short-term use [43]. This pattern suggests that benzodiazepine use was often recurrent and, for a subset of individuals, indicative of sustained or long-term use. In addition, the vast majority of benzodiazepine-related prescriptions were issued by non-psychiatric providers, with psychiatrists accounting for only 5.76% of all prescriptions. Consistent with previous studies, females [63] and older adults [58] in our sample were more likely to fill benzodiazepine prescriptions compared to males and younger adults. These findings underscore the need for policy and clinical measures to reduce inappropriate benzodiazepine use. Efforts should include greater access to psychotherapy and stricter regulation and monitoring of long-term use.

Participants reported high levels of life satisfaction, consistent with findings from previous research in Western countries where most individuals report being “slightly satisfied” to “satisfied” with life [14]. Many individuals with psychiatric diagnoses or filled psychotropic medication prescriptions also report being satisfied with life, although to a lesser extent than individuals without diagnoses and filled prescriptions. Higher anxiety and depressive symptoms, along with psychiatric disorder status, were associated with lower life satisfaction. Depressive symptoms had the greatest impact on life satisfaction, followed by anxiety symptoms, and, to a lesser extent, psychiatric disorder status. This suggests that the severity of symptoms, specifically depressive symptoms, may be more influential on satisfaction with life than the mere presence of a psychiatric disorder. The results are in line with evidence that psychiatric disorder status may have a small impact on satisfaction with life compared to subjective psychiatric health, which encompasses a broader range of factors than the mere absence or presence of a disorder [16,64]. Therefore, although individuals with a psychiatric disorder often report lower subjective psychiatric health, other aspects of psychiatric health may also impact life satisfaction [64]. Importantly, the findings support the idea that individuals can feel satisfied with life despite struggling with psychiatric health. Interestingly, females and older individuals were more likely to have a psychiatric diagnosis or to have filled prescriptions despite reporting greater life satisfaction. The higher life satisfaction reported by females and older adults, despite greater psychiatric disorder diagnoses and filled psychotropic medication prescriptions, may further indicate the role of coping strategies, resilience, and social factors buffering against reduced life satisfaction in these groups. Findings from a recent study demonstrated that differentiation of self (e.g., emotional reactivity and I-position) was important for mental well-being and life satisfaction, with resilience mediating the relationship between these dimensions and mental well-being among males, whereas social support played an especially important role among females [65].

The current study has several strengths. All Icelanders 40 years and older were invited to participate, and the high consent rate makes this, to our knowledge, among the most comprehensive nationwide population-based studies of adult psychiatric health conducted to date, integrating both medical records and self-report measures in a real-world setting. Further, the sample is representative of the Icelandic population 40 years or older [17]. The current study also has some limitations. The study included individuals aged 40 years or older, and since the onset of psychiatric problems was not assessed, the burden of psychiatric disorders that occur in childhood and adolescence could not be evaluated in the current study. In addition, although the sample is representative of the Icelandic population 40 years and older, non-responders to self-report measures (about 40%) were older than responders, had less education, were more likely to be retired, and had lower salaries. More females responded than males; however, the sex difference was much smaller among non-responders. Medical records only provide information on individuals who seek assistance for psychiatric health issues and do not include data on psychiatric diagnoses outside primary care or hospital settings, and since substance use disorders are not treated in these settings, rates might be underreported. Psychiatric inpatient rates were excluded because inpatient admissions can be assigned multiple ICD codes, obscuring the primary reason for admission. As a result, calculating the rate of individuals with a psychiatric ICD code during an inpatient admission would likely overestimate admissions for psychiatric disorders. Thereby limiting the accuracy of burden and treatment-need estimates. As aforementioned, an additional limitation is that psychiatric disorder diagnoses have not been validated in Iceland. Furthermore, rates of filled psychotropic medication prescriptions were not compared to prescribed and self-reported medication use, and therefore, discrepancies between prescribed, dispensed, and used medications could not be assessed.

Accurate, population-based psychiatric health data are essential for effective healthcare planning and public health policy. This study uses comprehensive methods to assess psychiatric health, combining healthcare registry data with self-reported symptoms and satisfaction with life. Results reveal the widespread burden of psychiatric disorders and extensive use among physicians of pharmacological treatment for psychiatric problems across the Icelandic population, emphasizing the need for evidence-based psychiatric health services. The predominance of non-specific, broad diagnostic categories reveals a classification issue that can cause difficulties in providing appropriate care. The high rates of filled psychotropic medication prescriptions point to medications being viewed as the primary solution to psychiatric symptoms and disorders, with a concerning number of individuals filling prescriptions without a formal diagnosis. Benzodiazepine prescriptions were notably high, given that clinical guidelines do not suggest this class of medications as first-line treatment due to negative side effects. The overreliance on psychotropic medications may, for example, stem from gaps in physician training and greater coverage of medication costs compared to psychotherapy. Together, the findings highlight the urgent need for precise diagnostic practices, policy changes to regulate and monitor benzodiazepine use, and increased access to first-line treatments for psychiatric disorders.

## Supporting information

S1 Table Participant demographics of responders and non-responders to self-report measures (The Generalized Anxiety Disorder Scale [GAD-7], The Patient Health Questionnaire [PHQ-9], and Satisfaction with Life Scale [SWLS]). The table presents descriptive characteristics of study participants, including mean age with standard deviation and frequency distributions of sex, birthplace, housing status, education level, employment status, monthly salary, psychiatric diagnoses, and filled psychotropic medication prescriptions. Percentages are based on available data for each variable. Continuous variables are presented as means and standard deviations and compared using t-tests; categorical variables are presented as frequencies and percentages and compared using chi-square (χ²) tests. ^a^*M* = mean of total scores; *SD* = standard deviation of total scores; *n* = number of participants. ^b^Information for age, sex, psychiatric diagnosis, and psychotropic medication was obtained from public registries and was available for all participants who registered for the study. ^c^Information on birthplace, housing, education, employment status, and salary is only available for part of the sample. ^d^Not mutually exclusive categories. ^e^Salary per month was converted from the Icelandic krona to Euros based on currency exchange rates on the 12th of April 2019, which is the median date of data collection. ***p < 0.0001. **p < 0.001. *p < 0.01.
(DOCX)

S2 Table Frequencies and percentages of individuals with a psychiatric diagnosis within the preceding year. The table presents the number and percentage of participants with the psychiatric disorder diagnosis in the year preceding study enrollment, categorized by ICD-10 diagnostic groups (F00–F99). Numbers are shown for the total sample and stratified by sex. Percentages are based on the total number of study participants (*n* = 80,733). ^a^ICD = International Classification of Diseases version 10. ^b^*M* = mean of total scores; *SD* = standard deviation of total scores; *n* = number of participants. ^c^Numbers are based on those with at least one psychiatric diagnosis in the past year.
(DOCX)

S3 Table Frequencies and percentages of individuals using psychotropic medications within the preceding year. The table presents the number and percentage of participants who filled a prescription for psychotropic medications within the year preceding study enrollment, categorized according to the Anatomical Therapeutic Chemical (ATC) classification system. Numbers are shown for the total sample and stratified by sex. Percentages are based on the total number of study participants (*n* = 80,733). The average number of psychotropic medications filled is also presented for the total sample and by sex. ^a^ATC = The Anatomical Therapeutic Chemical (ATC) Classification System.^b^*M* = mean of total scores; *SD* = standard deviation of total scores; *n* = number of participants. ^c^The average is based on those using at least one psychotropic medication in the past year.
(DOCX)

## References

[1] GBD 2019 Mental Disorders Collaborators. Global, regional, and national burden of 12 mental disorders in 204 countries and territories, 1990–2019: a systematic analysis for the Global Burden of Disease Study 2019. Lancet Psychiatry. 2022. doi: 10.1016/S2215-0366(21)00395-3 PMC8776563 35026139

[2] Institute for Health Metrics and Evaluation (IHME). Mental health: overview of burden and prevalence. Seattle (WA): IHME; 2024. https://www.healthdata.org/research-analysis/health-topics/mental-health

[3] Wright E, Pagliaro C, Page IS, Diminic S. A review of excluded groups and non-response in population-based mental health surveys from high-income countries. Soc Psychiatry Psychiatr Epidemiol. 2023;58(9):1265–92. doi: 10.1007/s00127-023-02488-y 37212903 PMC10423166

[4] Momen NC, Lasgaard M, Weye N, Edwards J, McGrath JJ, Plana-Ripoll O. Representativeness of survey participants in relation to mental disorders: a linkage between national registers and a population-representative survey. Int J Popul Data Sci. 2022;7(4):1759. doi: 10.23889/ijpds.v7i4.1759 37152406 PMC10161967

[5] World Health Organization. Global status report on alcohol and health and treatment of substance use disorders. Geneva; 2024. https://iris.who.int/server/api/core/bitstreams/32b161e9-5683-40f5-a1c3-1c92a76d5cda/content

[6] United Nations Office on Drugs and Crime. World drug report 2023. Vienna: United Nations; 2023.

[7] World Health Organization. Global status report on the public health response to dementia. Geneva: World Health Organization; 2021.

[8] Asselmann E, Beesdo-Baum K, Hamm A, Schmidt CO, Hertel J, Grabe HJ, et al. Lifetime and 12-month prevalence estimates for mental disorders in northeastern Germany: findings from the Study of Health in Pomerania. Eur Arch Psychiatry Clin Neurosci. 2019;269(3):341–50. doi: 10.1007/s00406-018-0911-5 29948253

[9] Viana MC, Kazdin AE, Harris MG, Stein DJ, Vigo DV, Hwang I, et al. Barriers to 12-month treatment of common anxiety, mood, and substance use disorders in the World Mental Health (WMH) surveys. Int J Ment Health Syst. 2025;19(1):6. doi: 10.1186/s13033-024-00658-2 39924481 PMC11807321

[10] Alonso J, Angermeyer MC. Psychotropic drug utilization in Europe: results from the European study of the epidemiology of mental disorders (ESEMeD) project. Acta Psychiatr Scand. 2004. doi: 10.1111/j.1600-0047.2004.00331.x 15128388

[11] Clery E, Morris S, Wilson C, Cooper C, Das-Munshi J, McManus S. Mental health treatment and service use. In: Adult Psychiatric Morbidity Survey: Survey of Mental Health and Wellbeing, England, 2023/24. London: NHS England; 2025.

[12] Maestre-Miquel C, López-de-Andrés A, Ji Z, de Miguel-Diez J, Brocate A, Sanz-Rojo S, et al. Gender Differences in the prevalence of mental health, psychological distress and psychotropic medication consumption in Spain: a nationwide population-based study. Int J Environ Res Public Health. 2021;18(12):6350. doi: 10.3390/ijerph18126350 34208274 PMC8296165

[13] Terlizzi EP, Norris T. Mental health treatment among adults: United States, 2020. 2020. 10.15620/cdc:110593 34672252

[14] Eurostat. Quality of life indicators – Overall experience of life. European Commission; 2024. https://ec.europa.eu/eurostat/statistics-explained/index.php?title=Quality_of_life_indicators_-_overall_experience_of_life

[15] Blanchflower DC, Bryson A. Life satisfaction in Western Europe and the gradual vanishing of the U-shape in age. Acad Ment Health Well-Being. 2025. doi: 10.20935/MHealthWellB7877

[16] Hinz A, Mehnert-Theuerkauf A, Glaesmer H, Schroeter ML, Tibubos AN, Petrowski K, et al. Changes in life satisfaction over six years in the general population: a longitudinal study with the Satisfaction With Life Scale (SWLS). PLoS One. 2025;20(1):e0316990. doi: 10.1371/journal.pone.0316990 39804846 PMC11729941

[17] Rögnvaldsson S, Love TJ, Thorsteinsdottir S, Reed ER, Óskarsson JÞ, Pétursdóttir Í, et al. Iceland screens, treats, or prevents multiple myeloma (iStopMM): a population-based screening study for monoclonal gammopathy of undetermined significance and randomized controlled trial of follow-up strategies. Blood Cancer J. 2021;11(5):94. doi: 10.1038/s41408-021-00480-w 34001889 PMC8128921

[18] Spitzer RL, Kroenke K, Williams JBW, Löwe B. A brief measure for assessing generalized anxiety disorder: the GAD-7. Arch Intern Med. 2006;166(10):1092–7. doi: 10.1001/archinte.166.10.1092 16717171

[19] Hinz A, Klein AM, Brähler E, Glaesmer H, Luck T, Riedel-Heller SG, et al. Psychometric evaluation of the Generalized Anxiety Disorder Screener GAD-7, based on a large German general population sample. J Affect Disord. 2017;210:338–44. doi: 10.1016/j.jad.2016.12.012 28088111

[20] Löwe B, Decker O, Müller S, Brähler E, Schellberg D, Herzog W, et al. Validation and standardization of the Generalized Anxiety Disorder Screener (GAD-7) in the general population. Med Care. 2008;46(3):266–74. doi: 10.1097/MLR.0b013e318160d093 18388841

[21] Beard C, Björgvinsson T. Beyond generalized anxiety disorder: psychometric properties of the GAD-7 in a heterogeneous psychiatric sample. J Anxiety Disord. 2014;28(6):547–52. doi: 10.1016/j.janxdis.2014.06.002 24983795

[22] Kroenke K, Spitzer RL, Williams JBW, Monahan PO, Löwe B. Anxiety disorders in primary care: prevalence, impairment, comorbidity, and detection. Ann Intern Med. 2007;146(5):317–25. doi: 10.7326/0003-4819-146-5-200703060-00004 17339617

[23] Ingólfsdóttir R. Psychometric properties of the Icelandic version of the Generalized Anxiety Disorder-7 (GAD-7) [BSc thesis]. Reykjavík: University of Iceland, Faculty of Psychology; 2014.

[24] Ólafsson EH. The psychometric properties of the Generalized Anxiety Disorder Scale (GAD-7) in a sample of older individuals from the Icelandic population. Reykjavík: University of Iceland, Faculty of Psychology; 2018.

[25] Harðardóttir D, Vésteinsdóttir V, Ásgeirsdóttir RL, Kristjánsdóttir H, Þórsdóttir F. Próffræðilegir eiginleikar íslenskrar þýðingar GAD-7 í klínísku úrtaki. Sálfræðiritið. 2022;27:25–38.

[26] Kroenke K, Spitzer RL, Williams JB. The PHQ-9: validity of a brief depression severity measure. J Gen Intern Med. 2001;16(9):606–13. doi: 10.1046/j.1525-1497.2001.016009606.x 11556941 PMC1495268

[27] Negeri ZF, Levis B, Sun Y, He C, Krishnan A, Wu Y, et al. Accuracy of the Patient Health Questionnaire-9 for screening to detect major depression: updated systematic review and individual participant data meta-analysis. BMJ. 2021;:n2183. doi: 10.1136/bmj.n2183 PMC8491108 34610915

[28] Levis B, Bhandari PM, Neupane D, Fan S, Sun Y, He C. Data-driven cutoff selection for the Patient Health Questionnaire-9 depression screening tool. JAMA Netw Open. 2024. doi: 10.1001/jamanetworkopen.2024.29630 PMC11584932 39576645

[29] Ágústsdóttir F, Daníelsdóttir S. The psychometric properties of the Patient Health Questionnaire (PHQ-9) in a sample of individuals 40 years and older from the Icelandic population. Reykjavík: University of Iceland, Faculty of Psychology; 2018.

[30] Björnsson AS, Jónsdóttir K, Sigurðardóttir S, Wessman I, Sigurjónsdóttir Ó, Þórisdóttir AS, et al. Próffræðilegir eiginleikar sheehan disability scale, quality of life scale og patient health questionnaire í íslenskri þýðingu. Icel J Psychol. 2018;23:91–100.

[31] Pálsdóttir VE. Réttmæti sjálfsmatskvarðans Patient Health Questionnaire (PHQ) gagnvart geðgreiningarviðtalinu Mini International Neuropsychiatric Interview (MINI) við að greina geðraskanir hjá heilsugæslusjúklingum. Reykjavík: University of Iceland; 2007.

[32] Kristófersdóttir KH. The validity of the PHQ-9 as a screener and to assess depression severity. Reykjavík: University of Iceland, Faculty of Psychology; 2020.

[33] Diener ED, Emmons RA, Larsen RJ, Griffin S. The satisfaction with life scale. J Pers Assess. 1985. doi: 10.1207/s15327752jpa4901_13 16367493

[34] Glaesmer H, Grande G, Braehler E, Roth M. The German version of the satisfaction with life scale (SWLS). Eur J Psychol Assess. 2011. doi: 10.1027/1015-5759/a000058

[35] Whisman MA, Judd CM. A cross-national analysis of measurement invariance of the Satisfaction With Life Scale. Psychol Assess. 2016;28(2):239–44. doi: 10.1037/pas0000181 26168309

[36] Durak M, Senol-Durak E, Gencoz T. Psychometric properties of the satisfaction with life scale among Turkish university students, correctional officers, and elderly adults. Soc Indic Res. 2010. doi: 10.1007/s11205-010-9589-4

[37] Pétursdóttir KE. Mat á próffræðilegum eiginleikum íslenskrar útgáfu Lífsánægjukvarðans. Reykjavík: University of Iceland, Faculty of Psychology; 2011.

[38] Unnarsdóttir AB, Gísladóttir KH, Ólafsdóttir RA. Psychometric properties of the Satisfaction With Life Scale (SWLS) in a sample of individuals over the age of 40 years from the Icelandic population. Reykjavík: University of Iceland, Faculty of Psychology; 2018.

[39] The Directorate of Health. Registers and health information. 2022. Accessed 2025 November 2. https://www.landlaeknir.is/english/registersandhealthinformation/

[40] Rögnvaldsson S, Long TE, Thorsteinsdottir S, Love TJ, Kristinsson SY. Validity of chronic disease diagnoses in Icelandic healthcare registries. Scand J Public Health. 2023;51(2):173–8. doi: 10.1177/14034948211059974 34903105

[41] Gudmundsson LS, Einarsson OB, Johannsson M. Icelandic medicines registry (IMR). In: Sturkenboom M, Schink T, eds. Databases for pharmacoepidemiological research. Cham: Springer; 2021. 205–11.

[42] RStudio Team. RStudio: Integrated Development Environment for R. 2025. Accessed 2025 November 2. http://www.rstudio.com/

[43] Brandt J, Bressi J, Lê M-L, Neal D, Cadogan C, Witt-Doerring J, et al. Prescribing and deprescribing guidance for benzodiazepine and benzodiazepine receptor agonist use in adults with depression, anxiety, and insomnia: an international scoping review. EClinicalMedicine. 2024;70:102507. doi: 10.1016/j.eclinm.2024.102507 38516102 PMC10955669

[44] Institute for Health Metrics and Evaluation. Global Burden of Disease Study 2019 (GBD 2019). Seattle (WA). 2019. https://ghdx.healthdata.org/gbd-2019

[45] Dattani S, Rodés-Guirao L, Ritchie H, Roser M. Mental health. 2023. Accessed 2024 May 1. https://ourworldindata.org/mental-health

[46] Stefánsson JG, Líndal E. The prevalence of mental disorders in the Greater-Reykjavik area. Laeknabladid. 2009;95(9):559–64. 19738288

[47] Substance Abuse and Mental Health Services Administration (SAMHSA). Key substance use and mental health indicators in the United States: results from the 2024 National Survey on Drug Use and Health. Rockville (MD): Center for Behavioral Health Statistics and Quality, Substance Abuse and Mental Health Services Administration; 2025. https://www.samhsa.gov/data/

[48] Institute for Health Metrics and Evaluation (IHME). GBD 2021 data input sources tool. 2024. Accessed 2024 October 28. https://ghdx.healthdata.org/gbd-2021/data-input-sources

[49] Stefánsson JG, Líndal E, Björnsson JK, Guðmundsdóttir A. Lifetime prevalence of specific mental disorders among persons born in Iceland in 1931. Acta Psychiatr Scand. 1991;84:142–9. 1950608 10.1111/j.1600-0447.1991.tb03118.x

[50] Kocalevent R-D, Hinz A, Brähler E. Standardization of the depression screener patient health questionnaire (PHQ-9) in the general population. Gen Hosp Psychiatry. 2013;35(5):551–5. doi: 10.1016/j.genhosppsych.2013.04.006 23664569

[51] European Institute for Gender Equality. Gender Equality Index 2021: Health. Luxembourg: Publications Office of the European Union. 2021. https://eige.europa.eu/publications-resources/toolkits-guides/gender-equality-index-2021-report/gender-differences-mental-disorders-begin-early-life

[52] Golinelli D, Sanmarchi F, Guarducci G, Palombarini J, Benetti P, Rosa S, et al. Gender differences in healthcare utilization across Europe: evidence from the European health interview survey. Health Policy. 2025;162:105448. doi: 10.1016/j.healthpol.2025.105448 41022014

[53] McDowell RD, Ryan A, Bunting BP, O’Neill SM, Alonso J, Bruffaerts R, et al. Mood and anxiety disorders across the adult lifespan: a European perspective. Psychol Med. 2014;44(4):707–22. doi: 10.1017/S0033291713001116 23721650

[54] Byers AL, Yaffe K, Covinsky KE, Friedman MB, Bruce ML. High occurrence of mood and anxiety disorders among older adults: the national comorbidity survey replication. Arch Gen Psychiatry. 2010;67(5):489–96. doi: 10.1001/archgenpsychiatry.2010.35 20439830 PMC2933177

[55] Tómasson K, Tómasson H, Zoëga T, Sigfússon E, Helgason T. Epidemiology of psychotropic medication use: comparison of sales, prescriptions and survey data in Iceland. Nord J Psychiatry. 2007;61(6):471–8. doi: 10.1080/08039480701773311 18236315

[56] Díaz-Gutiérrez MJ, Martínez-Cengotitabengoa M, Sáez de Adana E, Cano AI, Martínez-Cengotitabengoa MT, Besga A, et al. Relationship between the use of benzodiazepines and falls in older adults: a systematic review. Maturitas. 2017;101:17–22. doi: 10.1016/j.maturitas.2017.04.002 28539164

[57] Baek Y-H, Lee H, Kim WJ, Chung J-E, Pratt N, Kalisch Ellett L, et al. Uncertain association between benzodiazepine use and the risk of dementia: a cohort study. J Am Med Dir Assoc. 2020;21(2):201-211.e2. doi: 10.1016/j.jamda.2019.08.017 31653534

[58] Linnet K, Sigurdsson JA, Tomasdottir MO, Sigurdsson EL, Gudmundsson LS. Association between prescription of hypnotics/anxiolytics and mortality in multimorbid and non-multimorbid patients: a longitudinal cohort study in primary care. BMJ Open. 2019;9(12):e033545. doi: 10.1136/bmjopen-2019-033545 31811011 PMC6924757

[59] Maust DT, Lin LA, Blow FC. Benzodiazepine Use and Misuse Among Adults in the United States. Psychiatr Serv. 2019;70(2):97–106. doi: 10.1176/appi.ps.201800321 30554562 PMC6358464

[60] Santomauro DF, Purcell C, Whiteford HA, Ferrari AJ, Vos T. Grading disorder severity and averted burden by access to treatment within the GBD framework: a case study with anxiety disorders. Lancet Psychiatry. 2023;10(4):272–81. doi: 10.1016/S2215-0366(23)00037-8 36931778 PMC10017349

[61] Petitjean S, Ladewig D, Meier CR, Amrein R, Wiesbeck GA. Benzodiazepine prescribing to the Swiss adult population: results from a national survey of community pharmacies. Int Clin Psychopharmacol. 2007;22(5):292–8. doi: 10.1097/YIC.0b013e328105e0f2 17690598

[62] Nordic Medico-Statistical Committee (NOMESCO). Use of addictive medicine. Copenhagen (Denmark). 2023. https://nhwstat.org/health/thematic-articles-health/use-addictive-medicine

[63] Højlund M, Gudmundsson LS, Andersen JH, Saastamoinen LK, Zoega H, Skurtveit SO, et al. Use of benzodiazepines and benzodiazepine-related drugs in the Nordic countries between 2000 and 2020. Basic Clin Pharmacol Toxicol. 2023;132(1):60–70. doi: 10.1111/bcpt.13811 36314353 PMC10098719

[64] Lombardo P, Jones W, Wang L, Shen X, Goldner EM. The fundamental association between mental health and life satisfaction: results from successive waves of a Canadian national survey. BMC Public Health. 2018;18(1):342. doi: 10.1186/s12889-018-5235-x 29530010 PMC5848433

[65] Peleg O, Peleg M. Is resilience the bridge connecting social and family factors to mental well-being and life satisfaction?. Contemp Fam Ther. 2025;47(1):87–101.

