# Supplementary material for: Psychiatric health of Icelandic adults 40 years or older: A nationwide study of diagnoses, medications, and symptoms
Source: PLoS One. 2026 Apr 15;21(4):e0342075. doi: 10.1371/journal.pone.0342075 (PMC13082589; doi:10.1371/journal.pone.0342075)
Supplement: S1 Table — The table presents descriptive characteristics of study participants, including mean age with standard deviation and frequency distributions of sex, birthplace, housing status, education level, employment status, monthly salary, psychiatric diagnoses, and filled psychotropic medication prescriptions. Percentages are based on available data for each variable. Continuous variables are presented as means and standard deviations and compared using t-tests; categorical variables are presented as frequencies and percentages and compared using chi-square (χ²) tests. aM = mean of total scores; SD = standard deviation of total scores; n = number of participants. bInformation for age, sex, psychiatric diagnosis, and psychotropic medication was obtained from public registries and was available for all participants who registered for the study. cInformation on birthplace, housing, education, employment status, and salary is only available for part of the sample. dNot mutually exclusive categories. eSalary per month was converted from the Icelandic krona to Euros based on currency exchange rates on the 12th of April 2019, which is the median date of data collection. ***p < 0.0001. **p < 0.001. *p < 0.01. (DOCX) [file pone.0342075.s001.docx]

**S1 Table. Participant demographics of responders and non-responders to self-report measures (The Generalized Anxiety Disorder Scale [GAD-7], The Patient Health Questionnaire [PHQ-9], and Satisfaction with Life Scale [SWLS]).**

| Characteristic | *M (SD)*^a^*; Frequency (%)* | |  |
| --- | --- | --- | --- |
|  | Responders | Non-responders | *t-value; χ²* |
| Age^b^ | 56.0 (9.7) | 63.4 (12.1) | -97.3*** |
| Sex^b^ |  |  | 198.2*** |
| Female | 21,118 (56.8%) | 22,538 (51.8%) |  |
| Male | 16,097 (43.3%) | 20,980 (48.2%) |  |
| Birthplace^c^ |  |  | 1.1 |
| Iceland | 16,026 (97.3%) | 11,232 (97.1%) |  |
| Other | 444 (2.7%) | 337 (2.9%) |  |
| Housing^c^ |  |  | 6.3 |
| Homeowner, renting or  living with partner | 19,088 (97.5%) | 12,344 (97.1%) |  |
| Other | 483 (2.5%) | 373 (2.9%) |  |
| Education^c^ |  |  | 668.6*** |
| Some formal education | 404 (1.7%) | 430 (2.6%) |  |
| High school education | 4,041 (17.4%) | 4,030 (24.5%) |  |
| College education | 4,880 (21.0%) | 3,364 (20.4%) |  |
| Vocational college degree | 4,342 (18.7%) | 3,705 (22.5%) |  |
| University education | 9,545 (41.1%) | 4,937 (30.0%) |  |
| Employment status^c,d^ |  |  |  |
| Employed part or full-time | 17,119 (70.0%) | 9,951 (55.5%) | 951.2*** |
| Unemployed | 228 (0.9%) | 165 (0.9%) | 0.0 |
| Maternity leave | 15 (0.1%) | 5 (0.1%) | 1.8 |
| Student | 453 (1.9%) | 178 (1.0%) | 51.7*** |
| Homemaker | 481 (2.0%) | 448 (2.5%) | 13.3** |
| Sick leave | 445 (1.8%) | 311 (1.7%) | 0.4 |
| Disability | 1,927 (7.9%) | 1,365 (7.6%) | 1.1 |
| Retired | 4,846 (19.8%) | 6,058 (33.8%) | 1051.3*** |
| Salary^c,e^ |  |  | 671.1*** |
| Less than 1,110 | 342 (1.5%) | 307 (1.9%) |  |
| 1,111–2,220 | 3,413 (15.0%) | 3,695 (23.1%) |  |
| 2,221–3,700 | 6,391 (28.1%) | 5,056 (31.6%) |  |
| 3,701–5,190 | 5,782 (25.4%) | 3,384 (21.2%) |  |
| 5,191–6,670 | 3,440 (15.1%) | 1,755 (11.0%) |  |
| More than 6,671 | 3,377 (14.9%) | 1,791 (11.2%) |  |
| Psychiatric diagnosis^b^ | 7,090 (19.1%) | 9,674 (22.2%) | 123.0*** |
| Psychotropic medication^b^ | 11,491 (30.9%) | 16,151 (37.1%) | 346.2*** |

The table presents descriptive characteristics of study participants, including mean age with standard deviation and frequency distributions of sex, birthplace, housing status, education level, employment status, monthly salary, psychiatric diagnoses, and filled psychotropic medication prescriptions. Percentages are based on available data for each variable. Continuous variables are presented as means and standard deviations and compared using t-tests; categorical variables are presented as frequencies and percentages and compared using chi-square (χ²) tests.

^a^*M* = mean of total scores; *SD* = standard deviation of total scores; *n* = number of participants.

^b^Information for age, sex, psychiatric diagnosis, and psychotropic medication was obtained from public registries and was available for all participants that registered for the study.

^c^Information on birthplace, housing, education, employment status, and salary is only available for part of the sample.

^d^Not mutually exclusive categories.

^e^Salary per month was converted from the Icelandic krona to Euros based on currency exchange rates on the 12th of April 2019, which is the median date of data collection.

***p < 0.0001. **p < 0.001. *p < 0.01.
