# Supplementary material for: Psychiatric health of Icelandic adults 40 years or older: A nationwide study of diagnoses, medications, and symptoms
Source: PLoS One. 2026 Apr 15;21(4):e0342075. doi: 10.1371/journal.pone.0342075 (PMC13082589; doi:10.1371/journal.pone.0342075)
Supplement: S2 Table — The table presents the number and percentage of participants with the psychiatric disorder diagnosis in the year preceding study enrollment, categorized by ICD-10 diagnostic groups (F00–F99). Numbers are shown for the total sample and stratified by sex. Percentages are based on the total number of study participants (n = 80,733). aICD = International Classification of Diseases version 10. bM = mean of total scores; SD = standard deviation of total scores; n = number of participants. cNumbers are based on those with at least one psychiatric diagnosis in the past year. (DOCX) [file pone.0342075.s002.docx]

**S2 Table. Frequencies and percentages of individuals with a psychiatric diagnosis within the preceding year.**

|  | **Psychiatric diagnosis** | | |
| --- | --- | --- | --- |
| ICD code^a^ | Independent of sex | Males | Females |
|  | *n (%)*^b^ | *n (%)*^b^ | *n (%)*^b^ |
| F00−F03 | 255 (0.32%) | 126 (0.34%) | 129 (0.30%) |
| F04 | 1 (0.001%) | 0 (0.00%) | 1 (0.002%) |
| F05−F05.9 | 39 (0.05%) | 18 (0.05%) | 21 (0.05%) |
| F06−F06.9 | 174 (0.22%) | 75 (0.20%) | 99 (0.23%) |
| F07−F07.8 | 33 (0.04%) | 14 (0.04%) | 19 (0.04%) |
| F09 | 3 (0.004%) | 1 (0.003%) | 2 (0.005%) |
| F10 | 380 (0.47%) | 249 (0.67%) | 131 (0.30%) |
| F17 | 660 (0.82%) | 293 (0.79%) | 367 (0.84%) |
| F11−F16, F18−F19 | 188 (0.23%) | 86 (0.23%) | 102 (0.23%) |
| F20−F29 | 120 (0.15%) | 59 (0.16%) | 61 (0.14%) |
| F30−F30.9 | 39 (0.05%) | 14 (0.04%) | 25 (0.06%) |
| F31−F31.9 | 260 (0.32%) | 100 (0.27%) | 160 (0.37%) |
| F32 | 3,329 (4.12%) | 1,075 (2.90%) | 2,254 (5.16%) |
| F32.0 | 69 (0.09%) | 23 (0.06%) | 46 (0.11%) |
| F32.1−F32.11 | 101 (0.13%) | 34 (0.09%) | 67 (0.15%) |
| F32.2 | 62 (0.08%) | 25 (0.07%) | 37 (0.09%) |
| F32.3 | 4 (0.005%) | 2 (0.005%) | 2 (0.005%) |
| F32.8 | 10 (0.01%) | 5 (0.01%) | 5 (0.01%) |
| F32.9 | 2,919 (3.62%) | 938 (2.53%) | 1,981 (4.54%) |
| F33 | 368 (0.46%) | 118 (0.32%) | 250 (0.57%) |
| F33.0−F33.01 | 59 (0.07%) | 24 (0.07%) | 35 (0.08%) |
| F33.1−F33.11 | 144 (0.18%) | 46 (0.12%) | 98 (0.23%) |
| F33.2, F33.3 | 62 (0.08%) | 25 (0.07%) | 37 (0.09%) |
| F33.4 | 84 (0.10%) | 24 (0.07%) | 60 (0.14%) |
| F33.8 | 2 (0.003%) | 0 (0.00%) | 2 (0.005%) |
| F33.9 | 45 (0.06%) | 14 (0.04%) | 31 (0.07%) |
| F34 | 518 (0.64%) | 133 (0.36%) | 385 (0.88%) |
| F34.0 | 6 (0.007%) | 1 (0.003%) | 5 (0.01%) |
| F34.1 | 506 (0.63%) | 129 (0.35%) | 377 (0.86%) |
| F34.8 | 0 (0.00%) | 0 (0.00%) | 0 (0.00%) |
| F34.9 | 5 (0.006%) | 3 (0.008%) | 2 (0.005%) |
| F38−F38.8 | 1 (0.001%) | 0 (0.00%) | 1 (0.002%) |
| F39 | 6 (0.007%) | 1 (0.003%) | 5 (0.01%) |
| F40 | 206 (0.26%) | 64 (0.17%) | 142 (0.33%) |
| F40.0−F40.01 | 13 (0.02%) | 3 (0.008%) | 10 (0.02%) |
| F40.1 | 93 (0.12%) | 35 (0.09%) | 58 (0.13%) |
| F40.2, F40.8−F40.9 | 75 (0.09%) | 19 (0.05%) | 56 (0.13%) |
| F41 | 3,964 (4.91%) | 1,033 (2.79%) | 2,931 (6.71%) |
| F41.0 | 118 (0.15%) | 31 (0.08%) | 87 (0.20%) |
| F41.1 | 538 (0.67%) | 147 (0.40%) | 391 (0.90%) |
| F41.2 | 777 (0.96%) | 205 (0.55%) | 572 (1.31%) |
| F41.3 | 3 (0.004%) | 1 (0.003%) | 2 (0.005%) |
| F41.8 | 8 (0.01%) | 1 (0.003%) | 7 (0.02%) |
| F41.9 | 2,731 (3.38%) | 704 (1.90%) | 2,027 (4.64%) |
| F42−F42.9 | 59 (0.07%) | 21 (0.06%) | 38 (0.09%) |
| F43 | 1,465 (1.82%) | 350 (0.94%) | 1,115 (2.55%) |
| F43.0 | 72 (0.09%) | 19 (0.05%) | 53 (0.12%) |
| F43.1 | 201 (0.25%) | 43 (0.12%) | 158 (0.36%) |
| F43.2−F43.28 | 326 (0.40%) | 91 (0.25%) | 235 (0.54%) |
| F43.8−F43.9 | 94 (0.12%) | 18 (0.05%) | 76 (0.17%) |
| F44−F44.9 | 14 (0.02%) | 3 (0.008%) | 11 (0.03%) |
| F45−F45.9 | 45 (0.06%) | 18 (0.05%) | 27 (0.06%) |
| F48−F48.9 | 109 (0.14%) | 32 (0.09%) | 77 (0.18%) |
| F50−F50.9 | 19 (0.02%) | 0 (0.00%) | 19 (0.04%) |
| F51 | 9,171 (11.36%) | 2,990 (8.06%) | 6,181 (14.16%) |
| F51.0 | 37 (0.05%) | 12 (0.03%) | 25 (0.06%) |
| F51.1−F51.9 | 243 (0.30%) | 80 (0.22%) | 163 (0.37%) |
| F52−F52.9 | 557 (0.69%) | 552 (1.49%) | 5 (0.01%) |
| F53−F53.9 | 2 (0.003%) | 0 (0.00%) | 2 (0.005%) |
| F54 | 0 (0.00%) | 0 (0.00%) | 0 (0.00%) |
| F55−F55.9 | 31 (0.04%) | 16 (0.04%) | 15 (0.03%) |
| F59 | 0 (0.00%) | 0 (0.00%) | 0 (0.00%) |
| F60−F69 | 113 (0.14%) | 53 (0.14%) | 60 (0.14%) |
| F70−F79 | 53 (0.07%) | 19 (0.05%) | 34 (0.08%) |
| F80−F89 | 23 (0.03%) | 11 (0.03%) | 12 (0.03%) |
| F90−F98.9 | 365 (0.45%) | 151 (0.41%) | 214 (0.49%) |
| Total^c^ | 16,764 (20.77%) | 5,885 (15.87%) | 10,879 (24.92%) |
| Average number of psychiatric disorders^c^ | *M* = 1.91 (*SD* = 1.26)^b^ | *M* = 1.76 (*SD* =1.17)^b^ | *M* = 1.99 (*SD* = 1.30)^b^ |

The table presents the number and percentage of participants with the psychiatric disorder diagnosis in the year preceding study enrollment, categorized by ICD-10 diagnostic groups (F00–F99). Numbers are shown for the total sample and strati-fied by sex. Percentages are based on the total number of study participants (n = 80,733).

^a^ICD = International Classification of Diseases version 10.

^b^*M* = mean of total scores; *SD* = standard deviation of total scores; *n* = number of participants.

^c^Numbers are based on those with at least one psychiatric diagnosis in the past year.
