# Supplementary material for: Psychiatric health of Icelandic adults 40 years or older: A nationwide study of diagnoses, medications, and symptoms
Source: PLoS One. 2026 Apr 15;21(4):e0342075. doi: 10.1371/journal.pone.0342075 (PMC13082589; doi:10.1371/journal.pone.0342075)
Supplement: S3 Table — The table presents the number and percentage of participants who filled a prescription for psychotropic medications within the year preceding study enrollment, categorized according to the Anatomical Therapeutic Chemical (ATC) classification system. Numbers are shown for the total sample and stratified by sex. Percentages are based on the total number of study participants (n = 80,733). The average number of psychotropic medications filled is also presented for the total sample and by sex. aATC = The Anatomical Therapeutic Chemical (ATC) Classification System.bM = mean of total scores; SD = standard deviation of total scores; n = number of participants. cThe average is based on those using at least one psychotropic medication in the past year. (DOCX) [file pone.0342075.s003.docx]

**S3 Table. Frequencies and percentages of individuals using psychotropic medications within the preceding year.**

|  | ATC code^a^ | Independent of sex | Males | Females |
| --- | --- | --- | --- | --- |
|  |  | *n (%)*^b^ | *n (%)*^b^ | *n (%)*^b^ |
| Antipsychotics (N05A) | | 3,151 (3.90%) | 1,125 (3.03%) | 2,026 (4.64%) |
|  | Phenothiazines with aliphatic side-chain (N05AA) | 986 (1.22%) | 308 (0.83%) | 678 (1.55%) |
|  | Phenothiazines with piperazine structure (N05AB) | 157 (0.20%) | 56 (0.15%) | 101 (0.23%) |
|  | Phenothiazines with piperidine structure (N05AC) | 0 (0.00%) | 0 (0.00%) | 0 (0.00%) |
|  | Butyrophenone derivatives (N05AD) | 52 (0.06%) | 25 (0.07%) | 27 (0.06%) |
|  | Indole derivatives (N05AE) | 10 (0.01%) | 3 (0.008%) | 7 (0.02%) |
|  | Thioxanthene derivative (N05AF) | 286 (0.35%) | 116 (0.31%) | 170 (0.39%) |
|  | Diphenylbutylpiperidine derivatives (N05AG) | 5 (0.006%) | 3 (0.008%) | 2 (0.005%) |
|  | Diazepines, oxazepines, thiazepines, and ozepines (N05AH) | 1,700 (2.11%) | 605 (1.63%) | 1,095 (2.51%) |
|  | Benzamides (N05AL) | 29 (0.04%) | 13 (0.04%) | 16 (0.04%) |
|  | Lithium (N05AN) | 190 (0.24%) | 73 (0.20%) | 117 (0.27%) |
|  | Other antipsychotics (N05AX) | 234 (0.29%) | 95 (0.26%) | 139 (0.32%) |
| Anxiolytics (N05B) | | 9,165 (11.35%) | 2,820 (7.61%) | 6,345 (14.53%) |
|  | Benzodiazepine derivatives (N05BA) | 8,514 (10.55%) | 2,602 (7.02%) | 5,912 (13.54%) |
|  | Diphenylmethane derivatives (N05BB) | 797 (0.99%) | 247 (0.67%) | 550 (1.26%) |
|  | Azaspirodecanedione derivatives (N05BE) | 36 (0.05%) | 10 (0.03%) | 16 (0.04%) |
| Hypnotics and sedatives (N05C) | | 15,584 (19.30%) | 5,410 (14.59%) | 10,174 (23.31%) |
|  | Aldehydes and derivatives (N05CC) | 0 (0.00%) | 0 (0.00%) | 0 (0.00%) |
|  | Benzodiazepine derivatives (N05CD) | 815 (1.01%) | 315 (0.85%) | 500 (1.15%) |
|  | Benzodiazepine related drugs (N05CF) | 14,697 (18.21%) | 5,090 (13.73%) | 9,607 (22.01%) |
|  | Melatonin receptor agonists (N05CH) | 621 (0.77%) | 215 (0.58%) | 406 (0.93%) |
|  | Other hypnotics and sedatives (N05CM) | 76 (0.09%) | 44 (0.12%) | 32 (0.07%) |
| Antidepressants (N06A) | | 14,109 (17.48%) | 4,483 (12.09%) | 9,626 (22.05%) |
|  | Non-selective monoamine reuptake inhibitors (N06AA) | 3,072 (3.81%) | 704 (1.90%) | 2,368 (5.42%) |
|  | Selective serotonin reuptake inhibitors (N06AB) | 9,224 (11.43%) | 2,911 (7.85%) | 6,313 (14.46%) |
|  | Monoamine oxidase inhibitors, non-selective (N06AF) | 3 (0.004%) | 2 (0.005%) | 1 (0.002%) |
|  | Monoamine oxidase A inhibitors (N06AG) | 42 (0.05%) | 18 (0.05%) | 24 (0.06%) |
|  | Other antidepressants (N06AX) | 3,915 (4.85%) | 1,459 (3.94%) | 2,456 (5.63%) |
| Psychostimulants and nootropics (N06B) | | 811 (1.01%) | 355 (0.96%) | 456 (1.04%) |
|  | Centrally acting sympathomimetics (N06BA) | 809 (1.00%) | 354 (0.96%) | 455 (1.04%) |
|  | Xanthine derivatives (N06BC) | 2 (0.003%) | 1 (0.003%) | 1 (0.002%) |
| Antihistamines for systemic use | |  |  |  |
|  | Phenergan (R06AD02) | 1,508 (1.87%) | 490 (1.32%) | 1,018 (2.33%) |
| Total^c^ | | 27,642 (34.24%) | 9,660 (26.05%) | 17,982 (41.19%) |
| Average number of psychotropic medications^c^ | | *M* = 1.67  (*SD* = 0.99^b^ | *M* = 1.58  (*SD* = 0.93)^b^ | *M* = 1.72  (*SD* = 1.02)^b^ |

The table presents the number and percentage of participants who filled a prescription for psychotropic medications within the year preceding study enrollment, categorized according to the Anatomical Therapeutic Chemical (ATC) classification system. Numbers are shown for the total sample and stratified by sex. Percentages are based on the total number of study participants (*n* = 80,733). The average number of psychotropic medications filled is also presented for the total sample and by sex.

^a^ATC = The Anatomical Therapeutic Chemical (ATC) Classification System.

^b^*M* = mean of total scores; *SD* = standard deviation of total scores; *n* = number of participants.

^c^The average is based on those using at least one psychotropic medication in the past year.
